# Microbiota–gut–brain axis mechanisms in the complex network of bipolar disorders: potential clinical implications and translational opportunities

**DOI:** 10.1038/s41380-023-01964-w

**Published:** 2023-01-27

**Authors:** Miguel A. Ortega, Miguel Angel Álvarez-Mon, Cielo García-Montero, Óscar Fraile-Martínez, Jorge Monserrat, Lucia Martinez-Rozas, Roberto Rodríguez-Jiménez, Melchor Álvarez-Mon, Guillermo Lahera

**Affiliations:** 1 Department of Medicine and Medical Specialities, University of Alcala, Alcalá de Henares, Spain; 2 Ramón y Cajal Institute of Sanitary Research (IRYCIS), Madrid, Spain; 3 Department of Psychiatry and Mental Health, Hospital Universitario Infanta Leonor, Madrid, Spain; 4 Department of Legal Medicine and Psychiatry, Complutense University, Madrid, Spain; 5 Institute for Health Research 12 de Octubre Hospital, (Imas 12)/CIBERSAM (Biomedical Research Networking Centre in Mental Health), Madrid, Spain; 6 Immune System Diseases-Rheumatology, Oncology Service an Internal Medicine, University Hospital Príncipe de Asturias (CIBEREHD), Alcalá de Henares, Spain; 7 Psychiatry Service, Center for Biomedical Research in the Mental Health Network, University Hospital Príncipe de Asturias, Alcalá de Henares, Spain

**Keywords:** Bipolar disorder, Physiology

## Abstract

Bipolar disorders (BD) represent a severe leading disabling mental condition worldwide characterized by episodic and often progressive mood fluctuations with manic and depressive stages. The biological mechanisms underlying the pathophysiology of BD remain incompletely understood, but it seems that there is a complex picture of genetic and environmental factors implicated. Nowadays, gut microbiota is in the spotlight of new research related to this kind of psychiatric disorder, as it can be consistently related to several pathophysiological events observed in BD. In the context of the so-called microbiota–gut–brain (MGB) axis, it is shown to have a strong influence on host neuromodulation and endocrine functions (i.e., controlling the synthesis of neurotransmitters like serotonin or mediating the activation of the hypothalamic–pituitary–adrenal axis), as well as in modulation of host immune responses, critically regulating intestinal, systemic and brain inflammation (neuroinflammation). The present review aims to elucidate pathophysiological mechanisms derived from the MGB axis disruption and possible therapeutic approaches mainly focusing on gut microbiota in the complex network of BD. Understanding the mechanisms of gut microbiota and its bidirectional communication with the immune and other systems can shed light on the discovery of new therapies for improving the clinical management of these patients. Besides, the effect of psychiatric drugs on gut microbiota currently used in BD patients, together with new therapeutical approaches targeting this ecosystem (dietary patterns, probiotics, prebiotics, and other novelties) will also be contemplated.

## Introduction

### What are bipolar disorders?

**Fig. 1:**
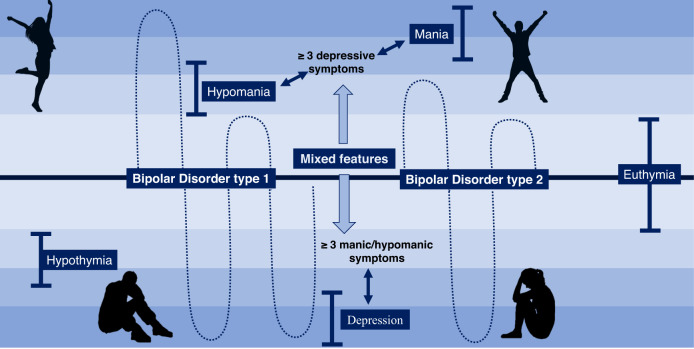
Characteristic fluctuations in two main types of bipolar disorder (BD). In BD type 1, patients experience at least one manic episode and various depressive episodes, whereas in BD type 2 they undergo at least one depressive episode and at least one hypomanic episode and have no history of manic episodes. A patient with type I or type II BD with “mixed features” consist of the co-occurrence either of hypomanic/manic episodes with 3 or more depressive symptoms or depressive episodes with 3 or more hypomanic/manic symptoms.

Bipolar disorders (BD) are a group of complex, severe, episodic and often progressive mood disorders considered as one of the leading causes of disability in the world. This cyclic disorder is distinguished by mood fluctuations, combining manic (bipolar mania), hypomanic and depressive phases (bipolar depression) [1]. Although a trimodal age‐at‐onset distribution has been proposed, this disease has typically its onset between adolescence and early adulthood [2]. According to Diagnostic and Statistical Manual of Mental Disorders V (DSM-V) [3] and International Statistical Classification of Diseases and Related Health Problems 11th (ICD-11) [4], BD can be classified into two main categories considering clinical features: BD type 1, when patients experience at least one manic episode and depressive episodes regularly, and BD type 2 when they undergo at least one depressive episode and at least one hypomanic episode and have no history of manic episodes [5]. Likewise, nowadays current clinical guidelines recognize that patients may exhibit episodes with “mixed features”. This term can be used for manic, hypomanic or depressive episodes in bipolar spectrum (type I and II) and major depressive disorders (MDD), characterize by the co-occurrence of three or more manic/hypomanic symptoms in a depressive episode or three or more depressive symptoms in a manic/hypomanic episode [6]. Recent evidence suggest that type 1 and type 2 BD should be considered as different entities. According to some studies, type 1 BD is associated with a more pronounced clinical presentation in both mania and depression [7], whereas type 2 BD cases are characterized by more marked and longer depressions with some hypomania and mixed features but not mania and rarely psychosis [8, 9]. They also had higher socioeconomic and functional status together with high levels of long-term morbidity and suicidal risk, hence denoting that both types are distinct, but not necessarily more or less severe than the others [8]. There is a third type of BD, cataloged as “substance/medication-induced bipolar and related disorder”, when those mood fluctuations are due to substance abuse or therapeutic drugs [1]. Schematically, these fluctuations can be visually understood through Fig. 1.

Epidemiologically, systematic review and meta-analysis found that BD type 1 has a pooled lifetime prevalence of 1.06% and 1.57% for BD type 2 [10], but numbers keep rising. On the other hand, in the last years, large sample studies have been analyzed reporting higher frequency in female patients in both types of BD [11, 12]. These studies argue that there must be sex differences as women have reported to present rapid cycling more often than men, besides the onset in female patients occurs with depressive phases and present more mixed manic presentations Moreover, BD can be accompanied by psychiatric comorbidities such as anxiety or substance abuse and impulse control disorders [13]. Not only BD may have devastating impact on patients but also an important economic burden for healthcare systems, with a range of direct costs per patient and per year oscillating from $881 to $27,617 (plus $1568–$116,062 indirect costs) [14]. On the other hand, this disease also involves high social stigma; one in four patients with BD reports high internalized stigma [15]. In relation to this fact, suicide affects 23–26% of BD patients, which represents about 3.4–14% of all suicide deaths [16], and sex-specific prevalence in this sense has also been observed, being relative suicide attempts higher in bipolar women, although less violent than men [11].

Nowadays, due to the complexity of this psychiatric disorder, there is neither molecular biomarker nor biological sign used for the diagnosis of BD in the clinical routine, so this entity remains a descriptive syndrome whose diagnosis is eminently clinical, based on the already mentioned clinical manuals such as the DSM-V and the ICD [17]. The pathophysiological bases of BD are not fully understood and deepen on the biological mechanisms underlying this intricate malady is an imperative need, as this could open potential translational applications.

### What is the pathophysiological basis of bipolar disorders?

To date, the etiopathogenesis of this disorder remains uncompleted and unclear, being plausible the hypothesis about the conjunction of both genetic, environmental and psychosocial factors. The heritability of BD seems to obey a complex non‐Mendelian inheritance [18] and some risk variants have been identified in previous works [19]. Thus, more than genes of major effect, evidence suggest that there are multiple susceptibility loci, each one with small effect. Furthermore, these loci seem to overlap with other psychiatric disorders, specially schizophrenia (SZ) [20]. Regarding environmental factors, compelling evidence support that they may influence the development of many psychiatric disorders, including BD. There is circumstantial evidence that certain environmental factors in pregnancy or in adulthood like viral infections could be associated with the clinical course of BD, although more evidence in this sense is warranted [21]. In the interface between genetics and environmental factors, epigenetics can be considered as the major biological mechanism explaining the etiopathogenesis of many psychiatric disorders [22, 23]. There are different epigenetic mechanisms involved in the regulation of DNA expression, including DNA methylation, histone methylation/acetylation and other modifications, along with non-coding RNAs such as micro RNAs (miRNAs) or long non-coding RNAs (lncRNAs) [24]. In the event of BD, obtained results are more limited than in other psychiatric disorders, although some authors argue that because of the nature of BD and its therapy, the field of epigenetics can be critical for understanding the clinical course of this complex disorder, aiding to define the role of environmental factors, and the possible identification of promising state and traits biomarkers [25].

**Fig. 2:**
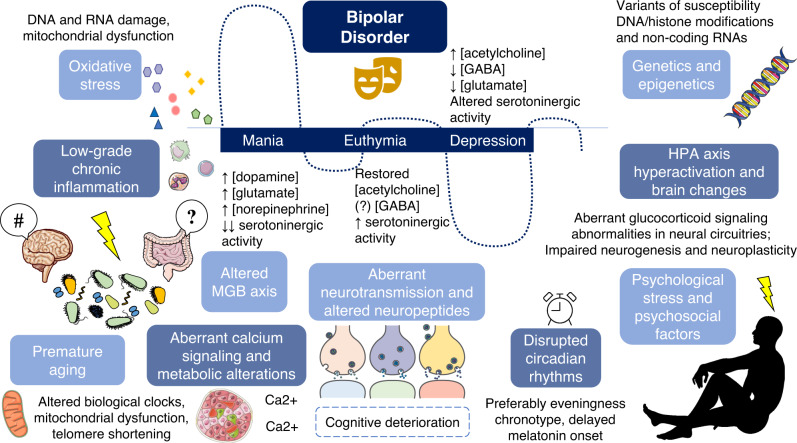
Network of a wide variety of factors involved in the etiopathogenesis of bipolar affective disorder (BD). Main factors described are differences in genetics (especially risk variants), epigenetics (DNA and histone modifications, non-coding RNAs), metabolic alterations; aberrant calcium signaling; alterations in circadian rhythms; oxidative stress, characteristic low-grade chronic inflammation, HPA axis hyperactivation, related to psychological stress and different psychosocial factors, accompanied with multiple changes in different neural circuitries, aberrant neurotransmission and altered neuropeptides. Peripheral blood concentrations of neurotransmitters are altered in BD, where in each phase there seems to be a different pattern of activity for every neurotransmissor. These differences are relevant with respect to healthy controls. Many of these events, together with additional mechanisms like altered biological clocks, mitochondrial dysfunction or telomere shortening can be considered as signs of premature aging. Beyond, all these factors influences and are directly influence by the dysregulation of the MGB axis which acts as a central element of psychiatric disorders. HPA hypothalamic–pituitary–adrenal, GABA gamma-aminobutyric acid.

From a molecular level, alterations in calcium signaling appear to be clearly associated with the development of BD [20, 26]. The intracellular calcium is essential for the modulation of several intracellular signaling cascades and neurotransmitter release [27]. Indeed, neurotransmitter dysregulation strongly underlies the pathophysiology of BD. Several neurotransmitters must be mentioned here, including monoamines (serotonin, dopamine and norepinephrine), glutamate, acetylcholine and gamma-aminobutyric acid (GABA). Importantly, the levels of these neurotransmitters can be different according to the phase of BD (Fig. 2) and can partially explain the cyclic fluctuations observed in these patients [28–35]. The aberrant neurotransmission is associated with several structural and functional changes in the brain and other encephalic structures, as it has been observed that patients with BD display abnormalities in neural circuitries supporting emotion processing, emotion regulation and reward processing [36]. These changes can also be different according to the history of these patients. For instance, image studies have found significant differences in brain activation of psychotic versus non-psychotic BD patients, showing that the former appears to be related to altered activation in left-sided regions whereas the latter exhibited right-sided functional alterations [37]. The brain of patients with BD are also characterized by impaired neurogenesis and neuroplasticity [38, 39]. In this sense, peripheral variations in some critical molecular markers involved in these processes such as brain-derived neurotrophic factor (BDNF) can be applied to understand the significant changes occurred in the brain, showing promising translational applications [40]. Alterations in different neuropeptides such as neuropeptide Y (NPY) and somatostatin have also been observed in patients with BD [41].

In addition, the role of oxidative stress markers in BD has been explored, as it seems that numerous lines of investigation of BD pathophysiology converge on oxidative stress and aberrations in oxidative energy generation. Although findings are not always steady, an increase in lipid peroxidation and in nitric oxide levels have been reported in BD patients, as well as increased damage in deoxyribonucleotide acid (DNA) and ribonucleic acid (RNA), compared with healthy controls [42]. Similarly, patients with BD exhibit telomere shortening which in turn can be associated with oxidative damage and other pathogenic mechanisms involved in BD [31]. Oxidative stress is also closely linked to mitochondrial dysfunction and despite findings are not consistent, it seems that deleted mitochondrial DNA is a manifestation in postmortem brains of patients with BD, which can be equally related to calcium dysregulation [43]. Even alterations in circadian rhythms seem to be implicated in BD [44], and it is thought that this fact potentiate episodes of mania and depression [45]. The dysregulation of sleep/wake cycle mediated by a delayed melatonin hormone onset reveals in most cases an eveningness chronotype [46] in these patients.

Similarly, hyperactivation of the hypothalamic–pituitary–adrenal (HPA) axis and aberrant glucocorticoid signaling is critically associated with this disorder. Although there is disagreement in affirming it is an etiological factor, what is undeniable is that this is a contributor to the BD clinical presentation and increases the risk of cognitive deterioration [47]. The hyperactivation of the HPA axis is closely related to the response of an individual to different psychosocial stressors. Among some of the most relevant psychosocial factors potentially related to BD, it must be highlighted cognitive dysfunction, altered domains of social cognition (theory of mind, emotion comprehension, empathy), autobiographical memory, temperament and personality factors (i.e. ruminative tendencies and neuroticism), which may drive to several difficulties in familial and social relationships [48]. Furthermore, there is a significant low-grade chronic inflammation reported in patients with BD, with an aberrant immune activation observed in the gut and systemically, which influences HPA axis, gut microbiota, metabolic functions, and the brain, driving to a phenomenon of neuroinflammation [49]. Likewise, dysregulation in the brain and systemic metabolism together with an altered hormonal profile represents another major feature of BD [50, 51].

As shown, the many factors involved in the etiopathogenesis of BD cannot be understood separately, but collectively exert a synergic effect, interacting bidirectionally. Beyond, many of the observed changes occurred in patients with BD can be considered as signs of premature aging (telomere length, oxidative stress, inflammation, disruptions on biological clocks, epigenetic aging and mitochondrial dysfunction), as prior works hypothesize that accelerated aging process is potentially implicated in the development and course of BD [52]. Likewise, another key branch to understand BD pathogenesis is the disruption of the so-called microbiota–gut–brain (MGB) axis [53–55]. Hence, the MGB axis can be considered a part of a great picture that influences and in turn is influenced by the different mechanisms involved in BD pathobiology. Because of that, there is a growing claim to study the gut microbiota and MGB axis as a potential biomarker for BD patients, offering promising clinical and translational opportunities [56]. Overall, a global picture of the plenty biological mechanisms involved in the etiopathogenesis of BD is summarized in Fig. 2.

### How is operating the microbiota–gut–brain axis in bipolar disorders?

Gut microbiota typifies a complex ecosystem consisting of trillions of microbes that inhabit the human intestine and which maintain a symbiotic relationship with their host [57]. This ecosystem represents about 2% of our body weight (approximately 1.5 kg) [58]. On the other hand, the available studies are also focused on gut microbiome, which refers not only to the different microbial populations that inhabit in the gut, but also their genome and end-products [59]. Bacteria are the most important component in gut microbiota, but also viruses—especially phages—Fungi, Amoebozoa or Archaea can be found. Among bacteria, Firmicutes, Bacteroides, Proteobacteria and Actinobacteria phyla represent over 90% of the gut microbiota community [60]. The shaping of gut microbiota starts at birth. The composition of gut microbiota differs according to delivery mode, so vaginal birth is preferable to cesarean section, which is linked with gut microbiota dysbiosis and even with subsequent repercussions in immunological and metabolic status [61, 62]. Successively, the gut microbiota is highly sensitive to environmental signals, receiving, integrating and responding to the information not only from the different organs of the body, but also from external influences like diet, physical activity, psychological and physical stress, sleep restrictions, socioeconomic status, drugs, antibiotics, exposure to pets, noise, and temperature [63]. In this sense, gut microbiota signifies a field of growing interest in the understanding of several processes in the organism regulating a plethora of metabolic routes, having a very close interplay with the immune system [60]. Because of the many functions that the gut microbiota fulfills, compelling evidence suggest that gut microbiota dysregulation could be directly associated with low-grade inflammation and a wide range of pathological conditions including obesity and metabolic disorders like type 2 diabetes [64, 65], inflammatory bowel disease (IBD) [66], gastrointestinal cancer [67] and psychiatric disorders, including BD [68].

There are several ways by which MGB axis works. A complex interplay between the gut microbiota, the parasympathetic nervous system—with a prominent role of vagus nerve-immune system and the different cells located in the gut is continuously occurring [69]. These interactions can occur by direct contact or indirectly through the secretion of a plenty of specific products and metabolites which influence the different parts of the MGB axis and the whole organism [70, 71]. The mucus layer of the gut is the scenario where most of the host-microbiota interactions take place. In these interactions, there is a basal and physiological inflammatory environment induced by gut microbiota that allows the regulation of bacterial populations, preventing its spread [71]. In this context, enterocytes communicate through innate immune receptors, chemokines and cytokines. For instance, Toll-like receptors (TLR) enable host innate immune system to identify pathogen-associated molecular patterns (PAMP) of microbes, such as lipopolysaccharide (LPS), lipoproteins or flagellin [72]. The brain receives and integrates signals from the gut directly from the afferent fibers of the vagus nerve [73] or due to the aforementioned products secreted by the different cells to the systemic circulation. In turn, the brain exerts a direct influence on the MGB axis through the efferent fibers of the vague nerve, or indirectly (i.e., by the activation of the HPA axis), denoting the bidirectional interplay occurred in this axis [74].

In this attractive and promising background, the aim of the present work is to study the relationships between BD and gut microbiota in the context of pathophysiology, and how the MGB axis interacts with the multiple systems and mechanisms behind this psychiatric condition. Equally, we will also collect the most relevant insights on the impact of BD pharmacotherapy on the gut microbiota, reviewing promising translational approaches targeting the MGB axis as well.

## Microbial ecosystem in patients with bipolar disorders

Gut microbiota composition of BD patients is different from healthy individuals, supporting its possible involvement in the pathogenesis of these complex entities [75]. BD, as many other psychiatric conditions, is related to a reduced microbial diversity and different relative abundance of bacterial phyla compared to controls [68]. Also, alterations in specific microbial populations are reported in these patients. For instance, Coello et al. [75] found that Flavonifractor, a genus linked to oxidative stress inducement, was associated with an odds ratio of 2.9 for having BD, but smoking could act as a potential confounder and more research is needed to confirm these results. Another study has also stated the existence of a potential causality between Betaproteobacteria and BD, associated with alterations in mucosal permeability and intestinal inflammation [76]. Oppositely, Aizawa et al. [77] failed to find any significant difference in the levels of Lactobacillus and Bifidobacterium between BD and healthy subjects; although their count appears to be directly correlated with sleep and serum cortisol levels. Besides, Lu et al. [78] reported that levels of *Faecalibacterium prausnitzii*, Bacteroides–Prevotella group, Atopobium Cluster, *Enterobacter spp*. and Clostridium Cluster IV were higher in BD patients than healthy subjects along with a reduced Bifidobacteria to Enterobacteriaceae ratio, having these changes a possible impact on brain function in these patients.

**Fig. 3:**
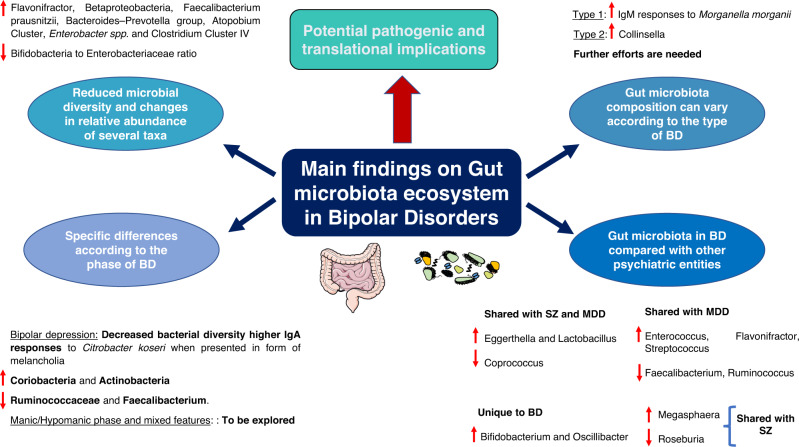
Main findings on gut microbiota ecosystem in bipolar disorders. As shown, BD patients are characterized by presenting a reduced microbial diversity and changes in relative abundance of several taxa, which may be involved in the pathogenesis of this complex disorder. Besides, specific differences according to the type and clinical manifestation, although further efforts are needed in hypomanic/manic phases, mixed features and also in the comparison between type 1 and 2 BD. Finally, compelling evidence is also supporting the notion that the gut microbiota can present some similarities and disparities across psychiatric disorders, opening the use of gut microbiota as potential biomarkers.

Interestingly, the differences in gut microbiota composition could partially explain clinical manifestations (types and phases) of BD. Notwithstanding there are no studies evaluating the gut microbiota composition in maniac episodes, it seems that the use of probiotics could prevent rehospitalization in patients who had suffered a recent episode of acute mania, thereby suggesting the involvement of gut microbiota in the development of maniac episodes [79]. In contrast, prior works have found that bacterial diversity differs in euthymic versus depressive phases in BD subjects, having this fact an epigenetic impact on the circadian clock gene ARNTL [80]. Despite the sample size being low (*N* = 32) and these results may be attributed to differences in their dietary patterns, the authors observed that these microbial changes appear to contribute to the pathogenesis of BD. Depressive stages in BD patients manifested in form of melancholia are associated with higher IgA responses to *Citrobacter koseri* than in healthy or non-melancholic depressed individuals [56]. Similarly, Painold et al. [81] found an inverse correlation between microbial alpha-diversity and illness duration in BD patients with a depressive episode, with increased levels of Coriobacteria and Actinobacteria together with decreased Ruminococcaceae and Faecalibacterium. Despite there is a lack of studies yet, we consider that future works could be directed to study the gut microbiota in episodes with “mixed features”, also focusing on the study of hypomanic/manic phases in order to establish and understand differences occurred in each phase (Fig. 3).

On the other hand, there are also some studies evaluating the gut microbiota composition in relation to the type of BD. Interestingly, there are works focused on comparing gut microbiota composition in patients with MDD, type I and type II BD. Notwithstanding there are some similarities in the MGB axis disruption in these three groups in comparison to healthy subjects, it seems that these aberrations are greater in type I BD patients and melancholia [56]. Indeed, individuals with type I BD showed higher IgM responses to *Morganella morganii* than patients with MDD and type II BD. Similarly, McIntyre et al. [82] reported that patients with type II BD exhibit a greater abundance of Collinsella in comparison to those with type I, although they noticed about the small sample size and insufficient control for some potential moderating factors like medication. More studies could be aimed to find if there is a differential gut microbiota profile in patients with type 1 versus type 2 BD (Fig. 3).

Also, some researchers have found in gut microbiota a promising point to compare and distinguish between BD and other psychiatric disorders. For instance, it seems that Prevotella 2 and Ruminococcaceae *UCG-002* are more prevalent in patients with MDD than BD patients [83]. Likewise, compared with healthy individuals, MDD is associated with alterations in Bacteroidaceae family, whereas disturbances in Lachnospiraceae, Prevotellaceae, and Ruminococcaceae families are related to BD [84]. Furthermore, the abundance of Fusobacteriaceae, *Escherichia blattae DSM 4481* and *Klebsiella oxytoca* were significantly augmented, whereas the *Bifidobacterium longum subsp. infantis ATCC 15697* = *JCM 1222* was significantly reduced in BD group compared with MDD group [85]. Besides, a very recent systematic review compared the gut microbiota in BD, MDD and SZ [86]. Interestingly, they observe that an increase in Eggerthella and Lactobacillus, together with a decrease in Coprococcus was common for the three conditions in comparison to controls. Likewise, MDD and SZ shared higher levels of Escherichia/Shigella and Veillonella whereas increased Megasphaera and lower Roseburia are common for BD and SZ. MDD and BD had more commonalities, including higher Enterococcus, Flavonifractor, and Streptococcus, and lower Faecalibacterium and Ruminococcus. They reported that each mental disorder could be potentially characterized by a different microbial profile: MDD was often characterized by higher Alistipes and Parabacteroides and lower Prevotella; BD by higher Bifidobacterium and Oscillibacter; and SZ by higher Prevotella and lower Bacteroides, Haemophilus, and Streptococcus [86]. Other studies have also found that BD and SZ are characterized by higher serum antibody levels to fungal pathogens *Saccharomyces cerevisiae* and *Candida albicans* and how these changes could be related to cognitive performance or the onset of psychotic symptoms [87].

Collectively, as shown in Fig. 3, these studies support the notion that there is an altered gut microbiota profile in patients with BD when compared to healthy subjects, and that changes observed in gut microbiota can correlate with the clinical manifestations, including both types and phases. Besides, by the analysis of gut microbiota in different psychiatric disorders the discovery of potential biomarkers that aid in the clinical diagnosis, prognosis or therapy prediction can also be opened. Due to the promising implications related to this field, we encourage further and more precise works to evaluate and study the gut microbiota in BD and other mental disorders.

## Microbiota–gut–brain axis in the context of bipolar disorder pathophysiology

To simplify, the pathophysiological mechanisms by which the MGB axis seems to influence the development of BD are (1) through modulating the enteric and central nervous system (CNS), with a focus on its immunomodulatory actions while influencing the intestinal, systemic and brain inflammation; and (2) because of the production of microbial metabolites with pleiotropic actions. In the following section, we will focus on the pathophysiological role of gut microbiota in BD patients.

### Gut and immune dysfunction in bipolar disorder

#### Intestinal epithelium disruption

The intestinal barrier constitutes the interface between the gut lumen and blood torrent, being crucial for the preservation of homeostasis. The gut harbors its own immune system, which is called intestine and gut-associated lymphoid tissue (GALT), key for whole body immune function. In physiological conditions, GALT should allow a tolerance of commensal bacteria and dietary antigens and act as a primary line of defense against luminal antigens and harmful substances in the host [88].

Intestinal barrier has varying degrees of permeability throughout the gastrointestinal tract. Zonulin modulates intercellular tight junctions and increases intestinal permeability in jejunum and ileum [89]. The increment of serum zonulin levels may be related to higher susceptibility for depression induced by stimuli [90], and zonulin and claudin-5 have seen increased in patients with BD [91]. Evidence has even found that levels of claudin-5 are associated with an earlier onset of BD, while reduced levels of this protein is associated with an extended duration of BD [92]. Prior studies suggest that there is an association between stress from early adverse life events and the development of irritable bowel syndrome (IBS), a functional disorder associated with a higher intestinal permeability [93, 94]. Increases in intestinal permeability have also been associated with autoimmunity diseases, inflammatory bowel disease (IBD) or celiac disease [95, 96]. Similarly, emerging evidence indicates that patients with mental disorders seem to present alterations of the gut microbiota and increased intestinal permeability [97, 98]. Specifically, IBS [99], celiac disease [100] and IBD [101, 102] have been associated with an increment in the risk of developing BD. In fact, both incidence and prevalence of psychiatric disorders (including BD) are higher in IBD patients [103].

#### Bacterial translocation

Gut microbiota can influence enterocyte junctions and therefore intestinal permeability [104]. When symbiotic relationship between gut microbiota and host is interrupted, dysbiosis leads to dysfunctions in mucosal barrier function, translocation of commensal microbes and chronic proinflammatory states [105, 106]. This disruption may have consequences CNS and immune response, potentially resulting in systemic disease [59, 107]. But, what is more; rising evidence supports that MGB axis establishes a bidirectional connection, so that central injury not only disrupts CNS, but also causes a gastrointestinal injury [108]. Furthermore, it has been well described that depression and other psychiatric disorders in which stress plays a key role, show alterations in gut microbiota, what consequently underpins translocation of bacterial products and triggers HPA axis activation [109]. Indeed, in BD bacterial translocation signs can be found. For instance, anti-*Saccharomyces cerevisiae* antibodies are higher in BD patients than in healthy controls, although these antibodies did not show to have an association with symptom severity or pharmacological treatment [110]. *Candida albicans* exposure has been associated with BD, especially in males and patients with somatic conditions [111].

An analyzable marker of bacterial translocation is the soluble cluster of differentiation (CD)-14; its levels are higher in BD patients compared with healthy individuals [112]. An association between soluble CD14 and anti-tissue transglutaminase IgG has also been described [112]. BD patients have increased levels of IgG antibodies to gliadin, especially during mania, compared to controls [113]. Further to this, the persistence of elevated antibodies to gliadin in patients with mania showed an association with rehospitalization rates in a 6-month follow-up [113]. Hence, bacterial translocation might be related to the clinical switch observed in these patients, although further studies are required in this field.

Moreover, microbial components can affect the intestinal barrier through diverse mechanisms. For instance, LPS is an endotoxin located in the outer membranes of numerous Gram-negative bacteria. This normally does not penetrate paracellular junctions because of its size, so that systems such as lipid rafts or clathrin-dependent mechanisms are necessary for LPS to penetrate into the cell [114]. Independently, when intestinal barrier is compromised, there is an increase in endotoxin in the blood torrent [114]. When LPS penetrates, the endotoxemia stimulates innate immune response, triggering systemic inflammation and aggravating neuroinflammation [115]. In this way, LPS levels can be used as a marker of bacterial translocation, presumably indicating that there is a weakened intestinal barrier, which is related to chronic systemic inflammation and insulin resistance [116, 117].

On the contrary, there are certain microbial products that modulate positively intestinal barrier function, besides, stimulating regulatory T CD4 cells (Tregs) and preserving intestinal epithelial lymphocytes [118]. These metabolites are short-chain fatty acids (SCFAs) among others and seem to be altered in BD as will be later discussed.

#### Intestinal inflammation and consequent systemic inflammation

Cytokines and chemokines are crucial for intercellular communication and contribute to maintain intestinal homeostasis through inflammatory mechanisms, but when these are constantly elevated, epithelial barrier integrity results compromised [119]. Proinflammatory cytokines such as Tumor Necrosis Factor (TNF)-α, interleukin (IL)-1β or IL-18 induce the endocytosis of epithelial apical junctional proteins [120], promoting an increment in intestinal permeability [121]. IL-1β is a key player in inducing intestinal inflammation; it increases epithelial tight junctions permeability, disrupting this barrier thorough the canonical NF-κB pathway [122]. Also, IL-18 can damage intestinal barrier, as it has been involved in tight junction modulation, mucosal apoptosis [123] and in inhibiting goblet cell maturation [124].

Altered levels in some of these cytokines have been observed in patients with BD, although more studies are needed in order to establish this relationship with intestinal permeability. Meanwhile, studies with varying results can be found in literature. Concretely, in a study with rapid cycling BD patients, IL-6 and IL-18 have been suggested as markers of manic episodes, due to significant levels in manic/hypomanic stages; however, IL-1 β was almost undetectable in plasma samples [125]. In another study, TNF-α, IL-6 and IL-18 were measured in serum from BD patients in manic, depressive and mixed state. The results concluded that TNF-α and IL-6 serum levels were significantly higher in manic, depressive and mixed-state patients compared with controls, but IL-18 was significantly higher only in depressive states. Nevertheless, it was alleged that confounding factors cannot confirm precisely the roles of these cytokines in the psychopathology of BD [126].

In general terms, systematic reviews of limited evidence affirm that it seems that TNF superfamily and proinflammatory cytokines contribute to the neuroprogression of the disease [127]. Moreover, what is known so far is that the abnormal immune response influence all stages of the disease, and potentially explains the elevated rates of comorbid inflammatory diseases found in these patients [128]. In fact, alterations can also be found in euthymia, when patients show to have greater levels of IL-8 in cerebrospinal fluid and monocyte chemoattractant proteins [129]. In patients with BD, stress-related neuroendocrine responses seem to be diminished while there is an increase in immune activation, related to incapability in reducing NF-κB and MAPK signaling [130].

Furthermore, during manic and depressive states, an elevated inflammatory signaling can be found; phospholipase A2 activity increases -liberating more arachidonic acid-, as well as levels of C-reactive protein, compliment and proinflammatory cytokines, compared to healthy individuals [131]. These alterations are attenuated in euthymia and with pharmacological treatment [131]. In relation to this, IL-6 in cerebrospinal fluid is significantly higher in patients with suicide attempts than in healthy controls, especially in violent attempts [132]. IL-13 levels have also been seen higher in euthymia and in mania [133]. Other findings also indicate that IL-6 and CRP levels are significantly higher in unipolar mania than in BD [134]. All these studies denote that BD is a group of complex mood disorders in which not only MGB axis is the central element. Evidence in recent years is demonstrating that elevated levels of peripheral proinflammatory signals are observed during all phases of BD, and that patients with autoimmune diseases present an increased risk of BD diagnosis.

Both innate and adaptive immunity play a prominent role in pathophysiology. Concretely, T helper-1 cells (Th1), which are IL-2, IL-6 and TNF-α producers, are hyperactivated in patients with BD; and also, a hyperactivation of Th2 has been associated with BD although more studies are required to elucidate clear links [135]. Also, some studies have exposed differences with MDD regarding immune cell populations. For instance, circulating levels of several subtypes of CD4+ Th cells, are higher in BD than in MDD including Th1, Th2, Th17 and Tregs [136]. However, there are contradictory results in other studies, as Treg populations are observed to decrease, but Th1 expansion is maintained as well as CD8+ cytotoxic T cell expansion [137]. All in all, intestinal integrity disruption and local and systemic inflammation go together with gut dysbiosis. The ecosystem harbored inside the intestine results damaged and, consequently, this increases inflammation (by bacterial components as described) and neuroinflammation, by neuroactive compounds (even from microbial metabolism) and other neuromodulatory mechanisms, as explained below.

#### Neuroinflammation and hypothalamic–pituitary–adrenal axis

Neuroinflammation consists of an inflammatory process within the brain or spinal cord, mediated by cytokines, chemokines, free radicals and other active ligands. Cytokines involvement in neurodegeneration has been discussed although it is not widely explained. A link between the elevated cytokines levels in CNS and in peripheral blood has been observed in postmortem studies of BD patients [138].

Low-grade systemic inflammation driven by several mediators previously debated, such as LPS, bacterial translocation and the increased proinflammatory cytokines and Th1 response, are all contributing to an immunological response in the brain [139]. A blood–brain barrier (BBB) dysfunction associated with BD is observed, also linked to gut microbiota, substance abuse and insulin resistance, all altering neuronal plasticity [140, 141]. BBB functions as a selective barrier that regulates the transport of molecules between blood and CNS, keeping necessities of nutrients and neurotransmitters in the brain. The dysregulation at this level enhances the activation of glial cells. Neuroinflammatory mediators are produced by these glial cells (microglia and astrocytes), besides endothelial and peripheral immune cells, all located in CNS [142]. Microglial hyperactivation induces damage to oligodendrocytes, affecting negatively myelination and neural circuits [143].

These disturbances would explain the findings in neuroimaging studies about white matter abnormalities in BD [144]. The inflammatory environment created by the release of cytokines by microglia in stressful situations seems to have a repercussion in behavioral phenotype in stress models, which is linked to neuropsychiatric disorders [145]. What is known so far is that cytokines-mediated neuroinflammation leads to dysregulations in the HPA axis, where the central regulator is cortisol releasing factor (CRF). Its hyperactivation produces an excess of cortisol, inducing depression-like behavior. This mechanism has been deeply studied in other psychiatric disorders like MDD [146]. As previously commented, this is consistent with the fact that so many patients with inflammatory-based diseases develop anxiety, MDD or BD [135]. Recent studies have found in BD a certain correlation between HPA axis dysfunction and the increased risk of relapses and cognitive impairments [47]. There is a growing body of evidence that suggest that dysfunctional microglia, oxidative damage, and mitochondrial dysfunction can play compelling roles in BD etiopathogenesis. Thus, an overactive neuroinflammatory response of astrocytes and microglia can contribute to BD development by alterations in immune response that leads to an increase in not only BBB integrity but also in intestinal permeability [147]. In fact, zonulin and claudin-5 have been proposed as biomarkers to detect BBB disruption [140] now that a diminished BBB integrity has been observed in patients with intestinal inflammation [148].

Although glucose is the principal energy fuel of the brain in normal conditions, lactate can be an alternative source during hypoglycemia and because of mitochondrial shifts in redox state or ischemia. Compared to healthy controls, higher levels of lactate from glycolysis in BD have been reported, which can relate to metabolic dysfunctions and a decrease in oxidative phosphorylation [149].

Abnormalities in mitochondria signaling lead to the production of free radicals like reactive oxygen species (ROS), which causes DNA damage, affecting neural plasticity and behavior [150]. ROS are produced in the brain mostly by microglia [32], and these products appear to be raised in patients with BD, which leads to oxidative and nitrosative damage. Indeed, dopamine, which is higher during mania, increases ROS, and it seems that, during mania and depression, there is a compensatory increase in antioxidant marker levels [151]. Furthermore, oxidative stress is associated with lower brain-derived neurotrophic factor (BDNF) levels [152]. This factor is crucial for the development, differentiation, plasticity, and survival of neurons, and it is decreased during mania and depression, but recovers at normal level in euthymic patients [32].

**Fig. 4:**
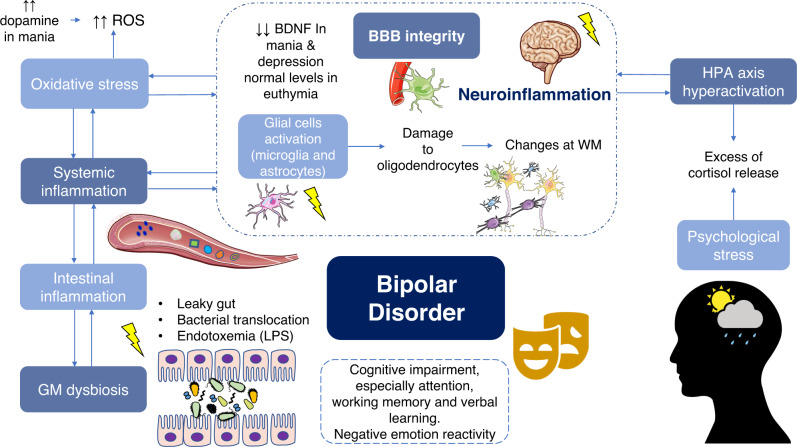
Factors involved in neuroinflammation in the context of bipolar disorder physiopathology. Leaky gut promotes bacterial translocation and consequent endotoxemia, what is a major contributor of inflammation, locally (intestinal) and systemically. Oxidative stress is also represented, and it seems to be even increased in certain phases; particularly, in mania, an increase in dopamine causes higher ROS production. Besides HPA axis hyperactivation is related to the aberrant control release in addition to psychological stress. Chronic inflammation causes impairment at BBB integrity, and then glial activation (microglia and astrocytes) is triggered. These mechanisms cause damage to oligodendrocytes, which causes imbalance at myelinization and therefore changes at WM. This cascade of events causes the so-called neuroinflammation, which promotes clinical manifestations of BD like cognitive impairment and negative emotion reactivity. ROS reactive oxygen species, BDNF brain-derived neurotrophic factor, BBB blood–brain barrier, WM white matter, HPA hypothalamic–pituitary–adrenal, GM gut microbiota, LPS lipopolysaccharide.

Sleep disruption may also aggravate this immune activation and neuroinflammation. Recent discoveries in animal models have demonstrated that some components from circadian system are mediators of microglial activation and neuroinflammation [153]. Mechanisms studied allege that immune activity and brain function are subjected to circadian clock machinery [154]. Moreover, sleep hygiene depends on the dynamics of the longest nerve in the autonomic nervous system, the vagus nerve—which controls the viscera—and also cerebral blood flow [155]. Chronic sleep disturbances have also been reported in BD. It is known that dysregulations in the circadian system can weaken cell antioxidant mechanisms, favoring an increased oxidative stress and lipid peroxidation, as there is a maintained damage from oxidative and nitrosative stress and higher levels of proinflammatory cytokines [156]. A consequence of this disruption resides in the abnormality of neurotransmitter release, related to HPA axis [157]. At the same time, stressful life events in patients with BD have been reported to be higher than in healthy people. These events affect vulnerability, onset and recurrence of BD, and increase cortisol levels [158], then following the process herein described in inverse direction. Figure 4 intends to summarize all these ideas.

Particularly, numerous BD patients manifest HPA axis hyperactivity in whole circadian rhythm, especially during mania [47]. Childhood trauma has been demonstrated to have a crucial role in HPA axis alterations in BD patients [159]. These patients showed to have increased levels of cortisol as well as lower glucocorticoid receptor’s function and alterations in glucocorticoid signaling. The hyperactivity of HPA axis is not the etiological factor that triggers BD, but undoubtable, it contributes to modify clinical presentations of BD and influence the genetic and environmental basic interaction in BD etiopathogenesis [47]. However, in patients with BD, neuropsychological performance is closely related to cortisol [160], and higher levels of this hormone have been related to poorer performance in some cognitive tasks [161, 162], especially working memory [163]. Moreover, abnormalities in this axis and in cortisol levels can be related to higher cardiovascular risk [164, 165], which may explain the increase in the mortality rate and in the cardiovascular risk factors prevalence observed in affective disorders, including BD [166].

Gut microbiota has bidirectional communication with HPA axis: certain microbial metabolites can attenuate it whereas translocated microbial antigens and exerted cytokines or prostaglandins productions activate it [95]. Moreover, dysbiosis status is associated with a constant increase in corticosterone synthesis in ileum, and, consequently, to glucocorticoid and insulin resistance, hyperglycemia and dyslipidemia [167]. This hypercortisolism is also associated with reduction in the immune response [97]. Then, HPA axis has an important impact on gut microbiota composition and neuroenteroendocrine functions, increasing gastrointestinal permeability, and contributing to the chronic low-grade inflammation [168]. Gut microbiota is not only a key player in regulating intestinal permeability, inflammation and neuroinflammation, but also in modulating behavior acting as an endocrine organ and modulating neural pathways through MGB axis as commented below.

### Neuromodulatory actions of gut microbiota in bipolar disorder

Gut microbiota can have important neuromodulatory activities either by direct actions (synthesizing or degrading neurotransmitters) or indirectly through the production of different microbial metabolites, which exert a plethora of systemic actions [169]. Besides, enteric and CNS can also influence neuromodulation actions of gut microbiota. In this section we will summarize the neuromodulatory implications of gut microbiota in patients with BD, focusing on its direct actions on neurotransmission and the main microbial metabolites (SCFAs, tryptophan metabolites).

#### Direct actions of gut microbiota on neurotransmission in bipolar disorder

As previously described, neurotransmitter dysregulation is a major pathophysiological signature of BD. Especially, GABA, glutamate, serotonin, dopamine, norepinephrine and acetylcholine appears to be importantly dysregulated in these patients [31, 170]. Gut microbiota is a central modulator of several neurotransmitters, being able to synthesize them and their precursors as well as promoting their degradation or metabolization to other products [171]. There are some species of bacteria grown in culture that can produce serotonin, although it seems that the most prominent role of gut microbiota in serotonin synthesis is achieved through the stimulation of enteroendocrine cells (EECs), especially by spore-forming bacteria [172]. The interplay between gut microbiota and EECs mostly occurs through different microbial metabolites, with a central role of SCFAs and tryptophan, as it will be later discussed.

Glutamate and GABA can be obtained from dietary sources or produced by the gut microbiota. Several bacteria are able of producing glutamate including Coryneform and lactic acid bacteria (LAB). Interestingly, glutamate also exert important modulatory effects on EECs, sending sensory signals to the vagus nerve [173] GABA is biosynthesized from glutamate due to the action of the enzyme glutamate decarboxylase, which is found in eukaryotic cells and in a broad spectrum of bacterial species [174]. However, LAB and more specifically certain Lactobacilli strains are the most important GABA producers in the gut [175]. In this sense, one study explored the possible role of Lactobacillus and Bifidobacterium in patients with BD. Interestingly, they show that notwithstanding the counts of Bifidobacterium or Lactobacillus were not significantly varied in patients with BD, it seems to exist a negative correlation between Lactobacillus and Bifidobacterium counts with sleep and cortisol levels respectively, being both important mechanisms in BD pathophysiology [77].

A broader number of bacteria have been reported to be able to produce other monoamines like dopamine and norepinephrine whereas only *Lactobacillus plantarum*seems to take part in the synthesis of acetylcholine [176, 177]. Despite the role of gut microbiota in the modulation of dopamine and norepinephrine remains to be fully understood, germ-free (GF) animal models have shown that there is a noteworthy alteration of these neurotransmitters in the gut as well as in the brain, which could have important consequences in the behavior [178].

Last but not least, nearly 117 types of bacteria have been identified as major histamine producers [179]. Apart from its well-known immunomodulatory role, histamine can act as a neurotransmitter in the brain, acting as a critical modulator of other neurotransmitters, and influencing arousal, motivation, and energy balance [180]. Neurons located in the posterior hypothalamus projects to all the major regions of the CNS, also participating in the regulation of sleep-wakefulness [181]. Likewise, the central histamine system in the brain is mediated via G-protein-coupled H1-H4 receptors and also appears to be involved in additional functions like arousal, control of pituitary hormone secretion, suppression of eating and cognitive functions [182]. Currently, there are some hypotheses supporting that patients with BD may have an altered histaminergic system, with upregulated histamine levels in manic phases and downregulated in depression, being their fluctuations closely related to other pathophysiological mechanisms [183]. An upregulated histamine production in the brain seems to drive to sleep-wake alterations in vivo, being associated with behavioral and metabolic disorders similar to those caused by voluntary sleep restriction in humans [184]. Conversely, animal models have also established that low levels of histamine-induced depression-like behavior, decreased locomotor activity in the home cage, and impaired aversive memory, leading to a decrease in wakefulness as well [185]. Hence, it is likely that the different alterations reported in the gut microbiota of patients with BD can be partially involved in the differential production of histamine and in turn this may be related to the described effects. Similarly, histamine production from gut microbiota appears to provide anti-inflammatory effects by suppressing TNF-α [186]. Thus, compelling evidence suggest the relevance of gut microbiota as pivotal player of brain function, influencing in the levels of several neurotransmitters and neuromodulators [187].

#### Short-chain fatty acids

SCFAs are end-products of saccharolytic fermentation of indigestible carbohydrates from our diet [188]. Also, proteins and peptides can be metabolized in the cecum and colon [189], as protein fermentation lead to branched SCFAs production [188]. The concentration of SCFAs is higher in cecum and proximal colon, and their levels decrease toward the distal colon [189]. The most important SCFAs are butyrate, propionate, and acetate, accounting approximately for 80% of the total, although the production of others like formate and valerate should also be highlighted [188–190]. Acetate and propionate are mainly produced by members of Bacteroidetes whereas butyrate is mainly synthesized by Firmicutes. Furthermore, butyrate can also be synthesized by the gut microbiota from acetate and lactate, although the last is not considered a SCFAs [191]. Notwithstanding mostly SCFAs uptake, signaling and functions occurring in the gut, associative studies show that SCFAs have an important role in human metabolism, the immune system and the entire organism, being able to cross the BBB [192, 193]. Critically, SCFAs are master epigenetic regulators, acting as inhibitors of the enzyme histone deacetylase (HDAC), which is involved in the histone modification [194, 195]. Thus, SCFAs are pleiotropic components produced by the gut microbiota involved in multiple processes of health and disease.

In this great context, butyrate is the most widely studied SCFA. Patients with BD show reductions in butyrate-producing bacteria which, being a central feature of MGB axis disruption [68]. In this sense, lower levels of Coprococcus, Faecalibacterium and Roseburia have been reported, while there seems to be an increase in bacteria populations that consume lactate, such as Megasphaera [86]. A recent systematic review has shown that despite the need for further studies, changes in butyrate-producer bacteria like Faecalibacterium may characterize BD in both a trait and state-dependent fashion [196]. In the gut, colonocytes consume butyrate locally, and this SCFA is crucial for maintaining the intestinal barrier [197]. SCFAs also stimulate enteroendocrine cells (EECs), promoting serotonin production in the colon [198]. This augmented production of serotonin leads to enhanced levels of serotonin in systemic circulation and in the brain, also stimulating vagal pathways and similar events occur with GABA, glucagon-like peptide 1 (GLP1) and peptide YY (PYY) [190].

Besides, immune cells have a high expression of SCFAs receptors, and it seems that SCFAs can regulate the function of colonic Treg cells [189, 199]. Despite butyrate and propionate seem to have the greatest immunomodulatory properties, all SCFAs can regulate the immune response [200]. Mostly, SCFAs exert anti-inflammatory properties. They induce the production of IL-10, closely related to intestinal homeostasis [201], and the production of IL-22 by innate lymphoid cells (ILC) requires the presence of gut microbiota IL-22 is not only crucial in intestinal homeostasis and mucosal barrier function, but also in host defense against infections [202]. Interestingly, most of the pharmacological treatment used in BD showed to increase IL-22, so the efficacy of these drugs may also be in relation with changes in this immune altered response [203]. Thus, reduced levels of butyrate and other SCFAs in patients with BD can have important consequences in the intestinal inflammation and permeability.

Moreover, SCFAs also have direct effects on the brain. For instance, SCFAs are associated with improved neuronal and cognitive function, BBB integrity and decreased neuroinflammation in the brain, representing a critical mechanism of interplay between gut microbiota and glial cells [190, 204]. Hence, despite more investigation is needed, it is conceivable that SCFAs can play a key role in the neural alterations observed in psychiatric disorders. The role of SCFAs in BD symptomatology has not been studied yet. However, a recent work analyzed SCFAs in fecal samples from 125 patients with psychiatric disorders (including 23 with BD). Interestingly, they found that self-reported depressive symptoms were positively associated with fecal acetate concentrations and negatively associated with butyrate and propionate levels [205]. Furthermore, there are in vivo studies showing that SCFAs can alleviate repeated psychosocial stress alterations, exerting antidepressant and anxiolytic effects [206]. Moreover, the therapeutic use of butyrate has been demonstrated to revert manic-like behavior in rats and regulates the antioxidant enzyme activities, protecting the brain against oxidative damage [207] therefore supporting the important role that SCFAs may play in the brain alterations in patients with BD.

Overall, there are plenty of potential effects of SCFAs in patients with BD, although more studies are required to unleash the microbial populations involved in this dysregulation. Furthermore, studying local and systemic levels of SCFAs would be of great aid to understand the pathophysiological basis of MGB axis disruption in BD, especially focusing on its different phases and types.

#### Tryptophan-kynurenine metabolism

Tryptophan is an essential amino acid with multiple metabolic fates and crucial in cell danger response, being again gut microbiota a modulator of its metabolic pathways [208]. Indeed, differences in tryptophan metabolizing bacterial pathways can be found in patients with neurological diseases [209] and psychiatric disorders including BD [210].

Gut microbiota can play a pivotal role in the metabolism of tryptophan. Five bacterial phyla including Firmicutes, Bacteroidetes, Actinobacteria, Proteobacteria, and Fusobacteria can participate in tryptophan metabolism, being those belonging to the Clostridium, Burkholderia, Streptomyces, Pseudomonas or Bacillus genera the most important modulators [209]. First, via hydroxylated pathway tryptophan is the precursor for serotonin or melatonin [176]. Thus, a large amount of serotonin is produced by enterochromaffin cells of the gastrointestinal tract [58, 208] and it is also present in enteric nerves [208]. Although tryptophan can cross the BBB, serotonin produced in the gut cannot [211]. However, serotonin can affect both vagus nerve and BBB permeability [69], and it modulates intestinal inflammation [212] and numerous physiological processes, including gastrointestinal secretion and peristalsis, vasoconstriction, behavior, and other neurological functions [177]. Besides, it can indirectly affect central serotoninergic pathways by modulating tryptophan and tryptamine availability. Thus, gut microbiota can play a central role in the serotoninergic dysfunction observed in patients with psychiatric disorders, although further studies are required.

The role of tryptamine in the brain is not well understood, although it is hypothesized that they may cross the BBB and exert important neurotransmitter or neuromodulatory actions [213]. Besides, increased tryptamine production can be associated with decreased circulating tryptophan and serotonin synthesis in the brain and could represent one mechanism by which tryptamine influence behavior [214]. Furthermore, tryptophan can be metabolized by gut microbiota to form indole and their derivates including indole-3-aldehyde, indole-3-acetic-acid and indole-3-propionic acid [176]. The role of tryptamine, indole and their derivate has been suggested in different psychiatric disorders, although little is known about their possible implication in BD [215].

On the other hand, tryptophan can be metabolized via the indoleamine 2,3-dioxygenase (IDO) [58, 176] or through the enzyme tryptophan-2,3-dioxygenase (TDO) to form N-formylkynurenine (Kyn) [216]. This is known as the Kyn pathway, and accounts for ~95% of dietary tryptophan degradation [217]. The indoles and their derivates can also be transformed into Kyn [58, 218]. Many components of the Kyn pathway are neuroactive, and influence neuroplasticity and/or exert neurotoxic effects; and this pathway is modulated by, and in turn modulates many other systems that are commonly disrupted in psychiatric disorders, including immune, endocrine, metabolic, and hormonal systems [219]. In a simple manner, Kyn can be metabolized either to kynurenic acid (KYNA), with neuroprotective properties, or to the neurotoxic components quinolinic acid (QA) and 3-hydroxykynurenine (3-HK) [208]. KYNA acts as an NMDA receptor antagonist or through the inhibition of the α7 nicotinic acetylcholine (α7nACh) receptors whereas QA acts as an agonist toward the NMDA receptors, thus promoting excitotoxic neuronal damage [220]. Regarding 3-HK, this molecule seems to exert a dual role in the CNS. On the one hand, it appears to induce oxidative damage and cell death, and high levels of 3-HK are associated with several psychiatric disorders. In contrast, some experimental studies have provided evidence of antioxidant and scavenging properties inherent to this component [221]. Howsoever, the synthesis of 3-HK from Kyn by the flavoprotein kynurenine-3-monoxygenase (KMO) is another critical point in the Kyn pathway, determining the balance between the neurotoxic component 3-HK and the neuroprotective KYNA [216]. Besides, it seems that Kyn and its downstream products exert pivotal immunomodulatory actions in the brain [222]. Thus, alterations in the Kyn pathway, with an augmentation of neurotoxic components like QA at the expense of neuroprotective derivates like KYNA, seem to have a central role in neuroinflammation in many neuropsychiatric disorders [223]. Likewise, systemic inflammation and glucocorticoids can also cross the BBB and influence the formation of QA in the brain by the microglia and infiltrated macrophages, enhancing the neuroinflammatory cascade [224]. Moreover, Kyn pathway may critically influence glutamate neurotransmission [225]. Thus, the gut microbiota can be a major modulator of Kyn pathway in different mental disorders, impairing tryptophan metabolism and influencing the brain and systemic inflammation [226]. Indeed, Kyn pathway seems to be abnormally activated in BD patients [227, 228]. Significant changes in different molecules of the Kyn pathway in BD patients have been described. For instance, meta-analysis on the peripheral blood levels of these components demonstrates that individuals with BD present lower peripheral blood levels of tryptophan, Kyn, KYNA, xanthurenic acid (a component derived from 3-HK), KYNA/Kyn and KYNA/QA ratio [229]. Besides, it seems that individuals with manic episode showed the greatest reductions in tryptophan levels whereas KYNA levels were more notably reduced among individuals in the depressive phase [229]. Other meta-analyses, however, despite showing that patients with BD present lower peripheral levels of tryptophan, Kyn or KYNA, did not find any significant differences between manic and depressed phases [230]. Intriguingly, it seems that this shift in the tryptophan metabolism from serotonin to the kyn pathway is associated with BD, MDD and SZ, but only in mood disorders (BD and MDD) there was a preferential metabolism of Kyn to the potentially neurotoxic QA [231], demonstrating the biological differences in this pathway between various psychiatric disorders. Interestingly, Kyn/tryptophan relation, which appears to represent IDO activity, is also elevated in BD patients [232]. Importantly, the increased IDO activity is a crucial mechanism related to the inflammation-induced depressive-like behavior by endotoxemia [233], thus showing the relevance that gut dysbiosis and bacterial translocation may have on BD patients. Van den Ameele et al. [234] observed that there was a strong relation between TNF-α and Kyn, Kyn/tryptophan, 3-HK and QA in the manic subgroup whereas in the depressed subgroup the KYNA/3-HK decreased and there was a strong association between interferon-y and Kyn pathway activation. Besides, both depressive and manic subgroups were characterized by presenting low levels of KYNA in comparison to healthy controls, which seems to support the relevance of the Kyn pathway in the context of neuroinflammation in BD and how it may be differentially modulated. Beyond, it seems that there is an inverse relationship between Oscillibacter (a genera increased in patients with BD) and the levels of tryptophan and KYNA, thereby supporting the relevance of gut microbiota in the altered Kyn pathway and neuroinflammation [235]. These findings could have important therapeutical implication for BD patients; as some antidepressant like ketamine can influence the Kyn pathway [227].

**Fig. 5:**
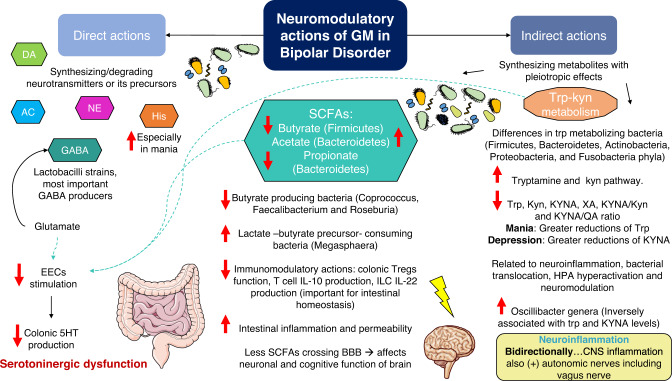
Summary of main neuromodulatory actions of gut microbiota (GM) in bipolar disorder (BD). There are direct (synthesis or degradation of neurotransmitters and their precursors) and indirect (synthesis of microbial metabolites with systemic pleiotropic effects) actions. In the indirect actions the main metabolites, SCFAs and tryptophan (trp) derived metabolites, are represented. Red arrows represent the result of pathological condition of BD, meaning the increased or decreased level in certain metabolic routes. Decreased levels of butyrate and propionate besides increased levels of acetate have been related to higher depressive symptoms. Tryptamine levels are risen whereas tryptophan levels are decreased in BD. Kynurenine pathway is also altered in BD and it seems to be related to neuroinflammation, bacterial translocation, HPA dysfunction, neuromodulation. As shown, a peripheral decrease in many metabolites of the Kyn pathway is shown, with specific differences in manic versus depressive stages and inversely correlated with certain gut microbiota populations (Oscillibacter genera). Glutamate concentration, abnormal SCFAs production and decreased trp, all confluence in less enteric serotonin (5HT) production due to decreased stimulation of enteroendocrine cells (EECs). All these mechanisms disruption contribute to bidirectional intestinal inflammation-neuroinflammation. Central nervous system (CNS) also sends signals activating vagus nerve. Studying local and systemic levels of SCFAs in patients with BD can be of aid to better understand microbiota–gut–brain (MGB) axis disruption in this malady. More studies focusing on its different phases and types are needed. This scenario is just the beginning of new therapeutical approaches. GM gut microbiota, DA dopamine, AC acetylcholine, NE norepinephrine, his histamine, GABA gamma-aminobutyric acid, EECs enteroendocrine cells, 5HT serotonin, SCFAs short-chain fatty acids, trp tryptophan, kyn kynurenine, KYNA Kynurenic acid, XA Xanthurenic acid, QA Quinolic acid, Tregs T regulator cells, ILC innate lymphoid cell, BBB blood–brain barrier, CNS central nervous system; (+): activates.

Taken together, this evidence suggests that gut microbiota plays a key role in BD. Gut microbiota is responsible for the disruption of intestinal permeability, which results in an upregulation of inflammatory cytokines. The gastrointestinal tract and brain maintain a crosstalk connection in which gut microbiota and inflammatory response are crucial, and this immune disruption is inextricably related to a neuroinflammatory response mediated by abnormalities in microglia and mitochondrial functions, as well as an increase in oxidative damage. CNS injury is a cause of inflammation, but this inflammation activates also autonomic nerves, including vagus nerve, feeding back this detrimental inflammation system. Aberrant neuroendocrine and immune responses seem to be critical in the precipitation and the feedback of neuroinflammatory-related disorders, such as BD. Neuromodulatory actions herein expressed in the section “Neuromodulatory actions of gut microbiota in bipolar disorder” are summarized in Fig. 5.

## Medical management of patients with bipolar disorders and the impact of pharmacological treatment on gut microbiota

Currently, the medical treatment of BD is eminently pharmacological, and it is based on antipsychotics, antidepressants, and other molecules such as lithium [18]. More detailly, mania should be treated first-line with lithium, divalproex, or an atypical antipsychotic medication; mixed episodes with first-line divalproex or an atypical antipsychotic; Bipolar depression treatment has limited evidence, although lots of drugs have been proposed as treatment options with similar efficacy but varying tolerability, such as lamotrigine, fluoxetine or antipsychotics as quetiapine or olanzapine, among others [236–238]. Despite the relevance of these clinical approaches, non-adherence rates under antipsychotics or mood stabilizers at long-term treatment in BD are high, with an estimation of about 40–50% [239]. In the non-adherence phenomenon, patient-related factors such as stigma or the knowledge about their illness, seem to be more influential than demographic or illness related variables [239]. Because of that, all patients should be offered individual, or group psychoeducation and a proper therapeutic drug monitoring could aid to inquire the effect of the treatment selected for each patient [238]. Similarly, electroconvulsive therapy (ECT) has proven to be a useful and safe option in severe and drug-resistant phases of BD episodes [240].

There is preliminary evidence that non-pharmacological therapies used in mental disorders like ECT are able to induce changes in gut microbiota [241, 242]. However, it has not been evaluated in patients with BD yet, and future studies can be focused on exploring the effects of this approach in this psychiatric condition. Conversely, compelling evidence points gut microbiota as a potential marker of pharmacological therapy in patients with BD [243]. Because of that, the field of “pharmacomicrobiomics*”* is gaining more attention in recent years, as it considers that both genetic and gut microbiota can influence the interindividual heterogeneity in drug responses [244]. Moreover, in a scoping review, the use of psychotropics for neurocognitive disorders, including BD, SZ, MDD or obsessive-compulsive disorder, could exert important antimicrobial effects on gut microbiota [210]. Thus, pharmacological treatment can have an important impact on gut microbiota, and in turn, gut microbiota can induce pharmacodynamical alterations by transforming drugs or by modifying host metabolism and immune system [244, 245]. Hence the bidirectional interaction between pharmacology and gut microbiota could be used as an important biomarker for patients with BD, allowing the identification of novel therapeutic targets or agents with better tolerability.

### Antipsychotics and gut microbiota

As aforementioned, antipsychotics are critical therapeutic agents for the clinical management of BD. Despite their benefits, weight gain, diabetes, metabolic syndrome, and cardiovascular disease are common side effects of this type of drugs, and insulin resistance that may be induced because of their use has been related to changes in DNA methylation, particularly with hypomethylation in fatty acyl CoA reductase 2 [246]. To this fact it must be added lipid and glucose dysregulation are common in BD patients, even before the diagnosis and treatment [247] and atypical antipsychotics can worsen these alterations. Previous works have demonstrated that atypical antipsychotics drive to notable alterations in gut microbiota populations, increasing the Firmicutes: Bacteroidetes ratio, fermentative metabolism as well as monosaccharide absorption or adipocyte fatty acid storage [248]. Thus, gut dysbiosis may be, at least partly, responsible for the metabolic dysfunctions induced by antipsychotics. Similarly, children and adolescents under chronic risperidone treatment showed a higher weight gain and, concurrently, changes in gut microbiota consisting in an altered ratio of Bacteroidetes: Firmicutes and an upregulation of metabolic pathways linked with weight gain, including alterations in butanoate, propanoate, fatty acid and tryptophan pathways [249]. In rats, olanzapine modifies gut microbiota to an obesogenic bacterial profile (similarly to the alterations observed in humans), so that gut microbiota is sufficient and essential to induce this weight gain [250]. Moreover, olanzapine not only acts synergistically with a high-fat diet, but it also proved to have antimicrobial activity in vitro against resident enteric bacterial strains [250]. Conversely, in rats, the coadministration of olanzapine and a purified prebiotic Bimuno™ galacto-oligosaccharides powder increased fecal Bifidobacterium and attenuated weight gain induced by olanzapine [251]. Also, the coadministration of olanzapine and an antibiotic combination consisting of neomycin, metronidazole and polymyxin B attenuated weight gain and metabolic dysfunction markers in rats [252]. Collectively, these studies appear to indicate that gut microbiota can be used as a promising therapeutic target to limit the development of different metabolic and cardiovascular risk factors observed in BD patients.

On the other hand, 4 weeks of treatment of quetiapine (300 mg/day) in patients with bipolar depression led to increased levels of *Eubacterium rectale*, Bifidobacteria, and Bifidobacteria to Enterobacteriacea ratio, having this fact a potential impact on brain function [78]. Another study conducted by Hu et al. [253] reported changes in 30 microbial markers after quetiapine treatment, and 10 of them presented an area under the curve (AUC) of 0.93 between responder and no responder patients, denoting a potential application of gut microbiota as a predictive biomarker.

Women under atypical antipsychotic treatment showed to have a decreased species diversity in their gut microbiota compared to healthy controls, while men did not show to have this difference [254]. Among these changes, a significant decrease in *Akkermansia muciniphila* was reported. This is a crucial Gram-negative bacterium capable of improving enterocyte layer integrity [255], whose levels decrease under high-fat diets and ageing [256]. The abundance of this strain has been inversely associated with inflammation markers, obesity, and metabolic disruption, including insulin resistance, cardiovascular risk parameters and adiposity [256], supporting again the possible role of gut microbiota in the metabolic adverse effects of antipsychotics. This information could be crucial, as BD is associated with a two-fold risk of suffering from cardiovascular disease [257] and we can find that changes in gut microbiota induced by certain drugs may be partly involved in the increased risk.

On the other hand, Cussoto et al. [258] found that aripiprazole, another atypical antipsychotic, was associated with a significant increase in the bacterial richness and diversity in mice. More detailly, aripiprazole induced an increase in *Firmicutes* phyla, *Peptostreptococcaceae, Clostridiaceae* and *Ruminococcaceae* family and in multiple minor genera such as *Clostridium sensu* stricto 1, *Ruminiclostridium 5, Intestinibacter, Eubacterium coprostanoligens, Peptoclostridium, Eubacterium oxidoreducens, Christensenellaceae uncultured and* Clostridia Family XIII, with a decrease in the relative abundance of *Ruminococcus* 1. Likewise, aripiprazole drive to an increase in the production of the SCFAs acetate and isovalerate. The role of isovalerate in psychiatric disorders is however uncertain, and there are some studies evidencing that an increase of this component is directly correlated with depression and cortisol levels, being able to interfere with synaptic neurotransmitter release after crossing the BBB [259]. Thus, further studies are needed to unravel the effects of antipsychotics on gut microbiota and how this may impact the response and side effects reported by the patients receiving this therapeutic regimen.

### Antidepressants and gut microbiota

Nowadays, neither monotherapy nor the use of antidepressants in rapid cycling BD is recommended, but antidepressants can be used as adjunctive treatment in BD as a second-line treatment. Thus, in BD type 1, serotonin reuptake inhibitors (SSRIs) and bupropion associated with lithium, divalproex or an atypical antipsychotic are reasonable options, while serotonin‐ norepinephrine reuptake inhibitors (SNRIs) and monoamine oxidase inhibitors (MAOIs) have a have higher propensity to induce manic switch and to produce mood destabilization. In BD type 2 sertraline and venlafaxine are preferred [13].

Moreover, the use of antidepressants has been contestable in BD because of the risk of manic switch or rapid cycling. In addition to this risk, it is important to highlight that antidepressants such as mirtazapine (especially associated with trazodone), fluoxetine, and nortriptyline have been associated with *Clostridium difficile* infection in depressed patients [260]. Moreover, some antidepressants seem to exert noteworthy antimicrobial effects, having been proposed that their effectiveness can be, at least partially, consequence of the effects on gut microbiota [261]. In turn, antimicrobials can also have antidepressant effects. However, further studies are needed to establish if the antimicrobial effects of antidepressants are associated with beneficial or detrimental mechanisms of these drugs, aiding to explain the therapeutic success or antidepressant resistance. For instance, SSRIs showed to have antimicrobial effects, mostly against Gram-positive microorganisms, acting as efflux pump inhibitors and having a synergistic activity when combined with some antibiotics. Sertraline, as a SSRIs, has an intrinsic antibacterial and antifungal activity in vitro, and it is capable of increasing antibacterial activities of antibiotics and making susceptible some previously resistant strains [262]. In murine models, fluoxetine, escitalopram, venlafaxine, duloxetine and desipramine showed to alter gut microbiota composition, mainly reducing Ruminococcus, Adlercreutzia and an unclassified Alphaproteobacteria [263]. Interestingly, *Ruminococcus flavefaciens* showed to alter the expression of genes linked with mitochondrial and neuronal processes in the medial prefrontal cortices, and the decrease of its levels could be associated with a relief in depressive-like behavior [263]. Thus, there is a promising line of research to explore the pharmacomicrobiomics of antidepressants in patients with BD and its clinical implications.

### Lithium and gut microbiota

Lithium is the mainstay of the treatment since it was discovered in 1949 for prophylaxis of BD [264]. This drug is used as an attenuator of calcium dysregulation, a critical mechanism involved in BD etiopathogenesis [20]. Although little is known about the effects of lithium on gut microbiota, it seems to favorably increase microbial species richness and diversity in vivo [258]. More precisely, at the phylum level, lithium appears to increase Actinobacteria and reduce Bacteroidetes, increasing the family of Peptostreptococcaceae, Clostridiaceae and Ruminococcaceae. Lithium also led to an increase in the relative abundance of *Clostridium sensu* stricto 1, *Ruminiclostridium 5, Intestinibacter, Eubacterium coprostanoligens, Peptoclostridium, Eubacterium oxidoreducens, Christensenellaceae uncultured and Clostridia Family XIII* and a decrease in the relative abundance of Bacteroides and Ruminococcus 1 [258]. Likewise, a very recent study showed that lithium carbonate was able to lighten colon inflammation by inducing changes in gut microbiota and increasing its diversity, especially expanding *Akkermansia municiphila* [243]. In this study, lithium could also activate anti-inflammatory Treg cell activity in lamina propria via metabolite-sensing G-protein coupled receptors 43 (GPR43), which mediates anti-inflammatory effects of the SCFA [243]. In relation to this fact, the immunomodulatory role of lithium in BD has been formerly discussed. Thus, chronic lithium therapy in euthymic BD patients can normalize immune parameters, leading to a decrease in cytokine-producing peripheral blood lymphocytes [265].

Despite the possible favorable effects of lithium on gut microbiota composition, the use of this drug has been associated with numerous withdrawals in patients with BD, including nausea, diarrhea, weight gain, fine tremor, hypothyroidism, electrocardiogram anomalies, alterations in renal function and even cognitive side effects [266]. This could be explained by two main alternatives: (1) these adverse outcomes can be independent of gut microbiota or (2) the use of lithium may have some unexplored effects on gut microbiota that can explain the occurrence of some adverse outcomes that could be used for therapy monitoring in patients receiving this medication.

### Anticonvulsants and gut microbiota

Anticonvulsants such as valproic acid, lamotrigine or carbamazepine are eligible therapeutical options in BD as mood stabilizers [267]. The effects of valproic acid from a biological perspective are multiple. On the one hand, some studies have described that valproic acid protects BBB function and integrity in a similar way to lithium [140]. On the other hand, valproic acid is detrimentally associated with metabolic anomalies, including higher levels of insulin and triglyceride, weight gain and increased body mass index [268], similar to antipsychotics. Considering its effects on gut microbiota, the study conducted by Cusotto et al. [258] also demonstrated that valproic acid raised bacterial diversity and richness in mice. This drug leads to an increase in Actinobacteria and Firmicutes phyla along with a decrease in Bacteroidetes. Likewise, valproic acid increase Peptostreptococcaceae, Clostridiaceae and Ruminococcaceae family, decreased the relative abundance of S24-7 uncultbact and enhance the relative abundance of *Ruminococcaceae uncultured, Clostridium sensu* stricto 1, *Ruminiclostridium 5, Intestinibacter, Eubacterium coprostanoligens, Peptoclostridium, Eubacterium oxidoreducens, Christensenellaceae uncultured* and *Clostridia* Family XIII. Also, according to this study, valproic acid administration induced a significant decrease in the levels of propionate and butyrate while augmenting the levels of isovalerate. Oppositely, in a murine model of valproic acid-induced autism, valproic acid administered to pregnant females significantly decreased the fecal microbiome diversity in pups, altering the composition of gut microbiota to resemble those derived from patients with autism spectrum disorder [269]. Similarly, the use of valproic acid at high doses used by patients can have noteworthy effects on the gut microbiota, altering the biosynthesis of fatty acid by different microorganisms [270]. Hence, more evidence is required to understand the favorable or detrimental effects of the use of valproic acid in the gut microbiota and its impact on BD.

On the other hand, lamotrigine does not have the metabolic adverse effects of, but it showed to have a temperature-dependent activity inhibiting ribosome biogenesis in *Escherichia coli* and *Salmonella enterica* [271], also displaying antimicrobial activity towards Gram-positive, such as *Bacillus subtilis* and *Staphylococcus aureus* [56]. Although there are few studies exploring the effects of carbamazepine on gut microbiota composition, carbamazepine also appears to exert notable antimicrobial effects, particularly on Gram-negative bacteria, inducing cytotoxicity in colonocytes [272]. This cytotoxic effect is shared with lamotrigine and appears to be contrary to the cytoprotective effects of certain microbial metabolites.

Nowadays, the implications of anticonvulsive therapy in gut microbiota remain unclear, but it seems to alter bacterial composition and metabolism. Future research might clarify the repercussion of these medications and if this interaction is meaningful or if it has any relation with its efficacy or adverse effects. The main findings collected about the impact of pharmacological treatment, its uses, side effects and consequences in gut microbiota are summarized in Table 1.

**Table 1:** Pharmacological treatment in bipolar disorder and its impact on gut microbiota.

Drug	Therapeutic effect	Clinical evidence	Side effects	Main findings on gut microbiota	References
Risperidone	Antipsychotic Anti-manic	Risperidone is one of the first-line treatment during acute mania. It could be also an option in maintenance treatment	Metabolic disruption (moderate risk): weight gain, diabetes, metabolic syndrome, insulin resistance and cardiovascular disease	Decreased *Akkermansia muciniphila* Decreased gut microbiota diversity in women Altered Firmicutes/Bacteroidetes ratio Modifications in butyrate, propionate and tryptophan pathways	[13, 246, 249, 251, 252, 254]
Olanzapine	Antipsychotic Anti-manic	Olanzapine is one of the second-line treatment during acute mania, but it is also effective in maintaining and preventing also depressive episodes in BD-1	Metabolic disruption (high risk): weight gain, diabetes, metabolic syndrome, insulin resistance and cardiovascular disease. These side effects position olanzapine as a second-line treatment	Decreased *Akkermansia muciniphila* Decreased gut microbiota diversity in women Increased Firmicutes/Bacteroidetes ratio In rats, olanzapine modifies gut microbiota to an obesogenic bacterial profile It also proved to have antimicrobial activity in vitro against resident enteric bacterial strains	[13, 246, 250, 254]
Quetiapine	Antipsychotic Antidepressant in BD	First-line treatment for both maintenance and acute depression in BD-1 and 2	Metabolic disruption (moderate risk): weight gain, diabetes, metabolic syndrome, insulin resistance and cardiovascular disease	Increased Firmicutes/Bacteroidetes ratio Decreased *Akkermansia muciniphila* Decreased gut microbiota diversity in women	[246, 248, 254]
Aripiprazole	Antipsychotic Anti-manic	First-line treatment in acute mania and maintenance in BD-1. First-line treatment in agitation	Metabolic disruption (very low risk)	Decreased *Akkermansia muciniphila* Decreased gut microbiota diversity in women Increase bacterial richness and diversity (increase in Firmicutes phyla, Peptostreptococcaceae, Clostridiaceae and Ruminococcaceae family and in minor genera like Clostridium sensu stricto 1, Ruminiclostridium 5, Intestinibacter, Eubacterium coprostanoligens, Peptoclostridium, *Eubacterium oxidoreducens*, Christensenellaceae uncultured and Clostridia Family XIII, with a decrease in the relative abundance of Ruminococcus 1) Increase acetate and isovalerate production	[246, 254, 258]
SSRIs	Antidepressant Antimicrobial	As a second-line treatment, SSRI can be used as an adjunctive treatment for acute depression in BD-1. Specifically, sertraline can be used as a second-line treatment for acute depression in BD-2	Antidepressant therapy may have a potential risk of inducing modifications or resistances in gut microbiota There is a risk of manic switch or rapid cycling, so they should not be used in monotherapy	Antimicrobial and antifungal properties have been described. Some antidepressants showed to reduce Ruminococcus, Adlercreutzia and an unclassified Alphaproteobacteria. Interestingly, lower levels of *Ruminococcus flavefaciens* can relieve depressive-like behavior The effectiveness of antidepressants could be related to their antimicrobial effects	[13, 260, 261, 263, 366]
Lithium	Anti-inflammatory. Mood stabilizer	Mainstay of the prophylaxis in BD (first-line treatment for maintenance in BD-1 and -2). It can also be used in bipolar depression (first-line treatment in BD-1 and second-line in BD-2). Commonly used in combination with other drugs	Nausea, diarrhea, Weight gain, fine tremor, hypothyroidism, electrocardiogram anomalies, alterations in renal function and cognitive side effects	Increase bacterial richness and diversity in mice (enhance Actinobacteria growth and reduce Bacteroidetes, increasing the family of Peptostreptococcaceae, Clostridiaceae and Ruminococcaceae and the relative abundance of Clostridium sensu stricto 1, Ruminiclostridium 5, Intestinibacter, Eubacterium coprostanoligens, Peptoclostridium, Eubacterium oxidoreducens, Christensenellaceae uncultured and Clostridia Family XIII decreasing the relative abundance of Bacteroides and Ruminococcus 1 Expands *Akkermansia municiphila* Modulates expression of a receptor that mediates anti-inflammatory effects of the SCFA	[13, 243, 258, 265, 266]
Valproic acid	Mood stabilizer Anticonvulsant	First-line maintenance treatment in BD-1. Second-line treatment in BD-1 depression	Metabolic anomalies, including higher levels of insulin and triglyceride, weight gain and increased body mass index	Increase bacterial diversity and richness in mice (increase Actinobacteria and Firmicutes phyla along with a decrease in Bacteroidetes; Promote the growth of Peptostreptococcaceae, Clostridiaceae and Ruminococcaceae family, decrease the relative abundance of S24-7 uncultbact and enhance the relative abundance of Ruminococcaceae uncultured, Clostridium sensu stricto 1, Ruminiclostridium 5, Intestinibacter, Eubacterium coprostanoligens, Peptoclostridium, Eubacterium oxidoreducens, Christensenellaceae uncultured and Clostridia Family XIII Valproic acid can alter the biosynthesis of the fatty acid productions in some gut microbiota species	[13, 258, 268, 270]
Lamotrigine and carbamazepine	Mood stabilizer Anticonvulsant	It can be used during bipolar depression (first-line treatment in BD-1 and second-line in BD-2) and as maintenance treatment (first-line treatment in BD-1 and 2)		Lamotrigine and carbamazepine induce cytotoxicity in colonocytes and present antimicrobial effects: Lamotrigine showed to have a temperature-dependent activity inhibiting ribosome biogenesis in *Escherichia coli* and *Salmonella enterica*, exerting antimicrobial effects against Gram-positive *Bacillus subtilis* and *Staphylococcus aureus* Carbamazepine has antimicrobial effects on Gram-negative bacteria	[13, 271, 272]

## Translational approaches modulating gut microbiota

Up to this point, it has been observed an important link between gut microbiota with several pathogenic mechanisms involved in BD. Likewise, the study of gut microbiota after treatment offers promising applications for therapy monitory or as biomarkers. On the other hand, a growing number of studies point MGB axis as a potential target of a broad spectrum of psychiatric disorders [63, 69, 187, 273]. In this section, we will collect the most relevant and updated knowledge related to the targeting of gut microbiota in BD patients. Nevertheless, it is important to notice that up to date there are no relevant conclusions drawn yet, as this type of therapy entails numerous challenges, such as the fact that gut microbiota is a dynamic structure continuously interacting with the inner and outer environment. Beyond, nowadays it has not been possible to identify a unique signature of a “healthy gut microbiota”, and the interindividual variation of this microbial ecosystem makes it hard to find adequate formulas or therapeutic approaches, critically determining the response of each patient to these interventions [274, 275].

### Dietary interventions

Diet is considered the greatest shaper of gut microbiota, as different dietary sources are needed for the biosynthesis of SCFAs and other microbial metabolites, determining the differential growth of certain bacterial populations [60, 276]. Besides, dietary patterns, food and nutrients exert pleiotropic effects in the entire organism, including in the MGB axis; being the gut microbiota a critical mediator of their benefits [277]. Thus, the modulatory effect of diet on gut microbiota can have in turn a direct influence on the immune system, the brain and the rest of the tissues in the body, regulating multiple biological processes epigenetically [23]. Because of that, dietary interventions have demonstrated their usefulness in multiple diseases characterized by an altered gut microbiota like mental disorders [278–280].

One of the most widely studied dietary interventions is Mediterranean Diet (MedDiet), characterized by a combination of high complex carbohydrates rich in fiber (vegetables, fruits, cereals and legumes), polyunsaturated fatty acids like omega-3 polyunsaturated fatty acids (PUFAs) or monounsaturated fatty acids (MUFA) with antiatherogenic and anti-inflammatory properties (found in olive oil, fish, seafood and nuts), and bioactive compounds found in plants with antioxidative properties such as flavonoids, phytosterols, terpenes and polyphenols. All these nutrients have beneficial effects on gut microbiota composition and function, contrary to westernized diets, abundant in refined carbohydrates (sugar or flour), low-quality fats (i.e., trans fatty acids), salt, additives and other detrimental components [71, 281]. In this sense, Łojko et al. [282] evaluated the dietary pattern followed by 113 euthymic BD patients in comparison with 160 healthy control subjects. Interestingly, they found that BD patients had lower Mediterranean Diet adherence than controls, showing unhealthy dietary patterns (western-type, pro-healthy carbohydrates, unhealthy snacks, and meats and potatoes). Likewise, they observed 70% of patients with BD had body mass index (BMI) >25 kg/m^2^ and they tend to present increased values of insulin resistance, with higher levels of fasting triglycerides, glucose index and waist circumference than the healthy subjects. Despite, they did not find any association between diet quality and the clinical course of BD, there is some preliminary findings supporting the role of diet in multiple biological mechanisms involved in BD, possibly influencing the clinical course and therapy success [279, 283]. The effects of diet on gut microbiota can be critically involved in this fact, representing a potential adjuvant therapy for BD and other mental disorders [284]. In this sense, a parallel randomized controlled trial also demonstrated that an isocaloric MedDiet in obese and overweight patients led to an increase in SCFA levels, Faecalibacterium strains (linked to butyrate metabolism) and in gene bacterial gene richness, diminishing *Ruminococcus gnavus* (with potentially proinflammatory properties) and decreasing systemic inflammation [285]. Other reported changes from MedDiet in gut microbiota include augmentation of Bacteroides, Lactobacilli, Bifidobacteria, Oscillospira, Roseburia, Clostridium cluster XIVa along with a reduction in Firmicutes and Proteobacteria phyla [286].

Of the many nutrients found in MedDiet, omega-3 polyunsaturated fatty acid (PUFAs) is perhaps the most widely studied, exerting the greatest benefits as an adjuvant for mental disorders [287]. In this line, a very recent systematic review [288] collecting results from 33 observational trials and 27 interventional studies established that dietary intake or supplementation of unsaturated fatty acids, mainly omega-3 PUFA seems to be associated with improved BD symptoms, together with seafood, folic acid and zinc. Nevertheless, omega-3 PUFA showed to improve depressive manifestations in BD, but not manic symptoms [289, 290] and seems to attenuate variability in mood, energy, irritability, and pain [291]. Intriguingly, some of the benefits of Omega-3 PUFA could be attributed to its modulatory role on gut microbiota. More detailly, omega-3 PUFA seems to modify its diversity by increasing the abundance of Bifidobacteria and Akkermansia as well as lowering Enterobacteria abundance [292]. Other reported changes include increase of Lactobacillus and Butyrivibrio, together with restoration of Firmicutes/Bacteroidetes ratio [293]. Moreover, they seem to enhance the mucosal barrier function and mitigate the inflammatory response linked with metabolic endotoxemia [292]. Conversely, some studies have found that nitrated dry cured meat has been associated with mania in humans, while alimentation with nitrated-added cured meat products leads to hyperactivity behavior (similar to mania) in rats [294]. These results were accompanied by increases in Lachnospiraceae and Erysipelotrichales abundance, two families linked to potential disruptions in fat and energy metabolism.

Overall, all these studies support that there is a possible, but still inconclusive role of diet in patients with BD, and these effects are partly mediated by gut microbiota. Further studies should deepen on the promising role of nutrition in the clinical course, monitoring or as an adjunctive therapy for these patients, especially for those with metabolic disturbances, overweight and unhealthy dietary patterns. Likewise, the recommendation and elimination of certain nutrients or foods, and nutritional education for these patients will surely bring notable benefits for patients with BD, being an additional part of the complex picture of this psychiatric malady.

### Prebiotics, probiotics and postbiotics

Probiotics are defined as: “live microorganisms, preferentially of human origin, that upon ingestion in specific and sufficient numbers confer unspecified health benefits to the host” [295]. For its part, prebiotics are “a selectively fermented ingredient that allows specific changes, both in the composition and/or activity in the gastrointestinal microflora that confers benefits upon host wellbeing and health” [296]. Dietary fibers are the principal prebiotics, and some examples of prebiotics include resistant starch, non-starch polysaccharides, inulin, and oligosaccharides such as fructooligosaccharides, galacto-oligosaccharides, and xylooligosaccharides [297]. These ingredients can resist hydrolysis in the human small intestine so they are fermented by colonic bacteria resulting in metabolites such as SCFA, whose importance has been previously discussed [297]. Pro- and prebiotics have numerous health benefits. Related to their favorable effect on gut microbiota, both seem to reduce inflammation, decrease some risk factors for cardiovascular disease, enhance satiety and promote weight loss and improve the bioavailability of minerals [298, 299]. Simultaneously, current evidence suggests that pro- and prebiotics seem to have anxiolytic and antidepressant effects in healthy population and in patients with mental disorders, improving psychological function via several mechanisms [300–302]. In this line, the term psychobiotic has been proposed as “a live organism that, when ingested in adequate amounts, produces a health benefit in patients suffering from psychiatric illness” [303].

The effects of probiotics in BD are not steady. Some researchers have not found significant differences in the severity of mania and depression in BD type 1 patients after probiotics supplementation [304]. In other studies, the probiotic supplementation of *Lactobacillus GG* and *Bifidobacterium lactis* strains showed to lessen the risk of rehospitalization in patients who were recently discharged from the hospital due to a mania episode. Interestingly, the benefits and efficacy of probiotic supplementation were significantly highly observed in patients with increased levels of systemic inflammation [79]. In a recent interventional study, 80 first-episode drug-naive patients with BD who received psychotropic therapy supplemented with either probiotic or placebo were followed up for 3 months for observing clinical symptom improvements and changes in oxidative stress markers [305]. After follow-up, decreased serum levels of lysophosphatidylcholines (LPCs) and increased serum levels of other six oxidative stress markers (creatine, inosine, hypoxanthine, choline, uric acid, allantoic acid) were observed in both placebo and probiotic groups. However, the adjuvant use of probiotics shows a positive correlation between changes in LPC (18:0) and Young Mania Rating Scale (YMRS scale), and in addition, the mania symptom greatly ameliorated in patients who received probiotic supplements as compared with the placebo. Moreover, dietary fiber, as natural prebiotic, combined with probiotics may be a useful therapeutic weapon to deal with the side effects of atypical antipsychotics [306]. In another intervention study, Reininghaus et al. [307] proved the benefits of probiotic supplementation on cognitive function in 20 euthymic individuals with BD over a time of 3 months. Interestingly, they found that this supplementation brought significant improvements to attention and psychomotor processing speed after 1 and 3 months of treatment while executive function seemed to improve 3 months after supplementation.

Overall, despite more studies are needed before drawing any conclusions, there are plenty of preliminary and promising evidence of the potential of probiotics (alone or in combination with prebiotics) for improving the clinical management of BD patients, as multiple studies have proven their benefits in mood, depressive symptoms and other mental health parameters [284, 308]. Because of the growing attention that is receiving, we also propose to drive further studies on the field of postbiotics, which can be defined as “a preparation of inanimate microorganisms and/or their components that confers a health benefit on the host” [309]. In this line, SCFAs represent the postbiotic most widely studied, particularly due to their epigenetic activity modulating histone acetylation and, consequently, gene expression. In bovine mammary epithelial cells, sodium propionate and sodium butyrate showed to reduce the activity of certain histone deacetylases as well as increase the acetylation of particular histones [310]. Thus, after the administration of LPS in bovine mammary epithelial cell, sodium butyrate showed to inactivate NF-κB signaling and, consequently, diminishing inflammatory response and apoptosis [311]. Notwithstanding the fact that more evidence is needed, this protective effect may represent a promising therapeutical alternative to modulate gut microbiota in BD. In rats, sodium butyrate showed to have an antimanic effect by attenuating the hyperactivity and the neurotrophic factors and metabolic disruption induced by ouabain, similar to mood stabilizers [207, 312, 313]. Because of the multiple mechanisms that postbiotic exert in the MGB axis [314], we expect that exploring the therapeutic use of postbiotics can represent an attractive line of research for future works.

### Other therapeutic approaches

Apart from diet, probiotics and prebiotics, there are interesting lines of research that could be used as valuable alternatives to consider for therapeutic studies.

#### Antimicrobials

As aforementioned, not only certain drugs used in BD exerted antimicrobial activity, but also antimicrobials could have noteworthy effects on mental functioning, because of their direct effect on gut microbiota. For instance, it has been suggested that tetracycline antibiotics might represent an attractive therapeutic alternative for different psychiatric disorders [315]. Minocycline is a tetracycline antibiotic that showed to increase neuronal survival, as well as being able to reduce proinflammatory cytokines production and modulating glutamate and monoaminergic pathways, which explains its anti-inflammatory and neuroprotective activity [316]. Aspirin and minocycline combination showed to be effective in BD depression, and minocycline was especially effective at higher baseline levels of IL-6, suggesting that both options could be evaluated as an adjunctive therapy [317]. Likewise, the use of minocycline can be particularly effective for the therapy of bipolar depression in patients with high glutathione (GSH) levels [318]. Similar roles have been given to another tetracycline, doxycycline, which appears to be a promising adjunctive therapy with lithium for treating BD, according to preclinical models [319]. Other studies and systematic reviews, however, did not find any benefits from using minocycline as adjunctive therapy in patients with BD [320, 321], and contrary to some proposals, doxycycline exposure in the adolescence did not report any causal relationship between this tetracycline and BD development [322]. Regarding the effects of minocycline treatment on gut microbiota, it seems that this therapy decreases the Firmicutes/Bacteroidetes ratio [323]. Likewise, changes in other genera have been reported under minocycline treatment, such as decreases in Allobaculum, Bifidobacterium, Turicibacter and Clostridium or increases in *Akkermansia muciniphilla*, Lachnospiraceae and *Ruminococcaceae incertae sedis* [324]. Regarding the effects of doxycycline on gut microbiota, previous studies have noticed an effect of this antibiotic on a short-term reduction of LAB like Bifidobacterium or Lactobacillus [325, 326]. On the other hand, there are some antibiotics that may be associated with the development of mania, what experts have designed as “antibiomania” [327]. According to a systematic review of 47 cases of antibiomania, 12 different antibacterial agents were implicated, with antitubercular agents, macrolides and quinolones being the most common causative groups [328]. Despite the pathophysiological basis of antibiomania remains to be fully unraveled, the suspected drug should be immediately discontinued and manic symptoms according to the clinical guidelines. Hence, despite some preliminary evidence is available supporting the use of antimicrobials in the clinical management of BD, further studies are warranted to evaluate the effects of antimicrobials in the gut microbiota and its association with clinical outcomes in these patients.

#### Fecal microbiota transplantation

Fecal microbiota transplantation (FMT) is another promising translational approach targeting gut microbiota. In a simple manner, FMT consists of transferring stool from a healthy donor into the colon of a patient with an established pathology with the aim to restore the normal microbiota and cure or at least ameliorate the disease [329]. Currently, FMT can be considered to treat recurrent *Clostridium difficile* infection, although there are multiple lines of research opened in intestinal and extraintestinal pathologies [330]. In the literature, there is a case report of a woman diagnosed with BD who went through FMT from her healthy husband at least nine times. Afterward, she had neither manic nor depressive symptoms in the following sixth months, also showing an important weight loss of nearly 33 kg [331]. However, despite these promising results, further studies are needed to evaluate FMT in patients with psychiatric disorders, as this field entails several challenges regarding donors and recipients [55]. Currently, there is a clinical trial (NCT03279224) conducted to assess the feasibility, efficacy, safety, and tolerability of FMT on patients with BD depression. The results of this trial are not available yet (on November 4, 2022), but in this workshop participants are randomized to receive either screened and processed donor stool (allogenic FMT) or their own stool (autologous FMT) via colonoscopy and monitored for 24 weeks post intervention. Then, depressive and manic symptoms, treatment acceptability, gastrointestinal and other side effects are assessed at baseline (prior to randomization) and weekly, whereas stool samples to evaluate microbiome composition are obtained at baseline and 3 and 6 months [332].

#### Immune-based approaches

Immune-based therapeutic strategies have been suggested for some patients with BD and MDD, although due to the biological and clinical heterogeneity of these complex disorders, this type of therapy could be considered as a part of personalized approach, beneficiating approximately one in three patients, according to previous studies [333]. Thus, some immunomodulatory drugs such as COX inhibitors exert not only anti-inflammatory outcomes but also antidepressant effects [334]. In this sense celecoxib showed to be a safe and a highly effective therapeutical option in resistant bipolar depression, reducing anxiety and accelerating treatment response [335], in a similar way to aspirin [336]. It should be highlighted that, although short-term administration of celecoxib does not seem to alter the bacterial abundance, relative changes in some bacterial populations have been described although changes in butyrate production are not observed after its administration [337]. TNF-α inhibitors have also shown antidepressant properties [338], so they have also been proposed as a potential treatment in BD with contradictory and unsatisfactory results [339]. For instance, in a study with BD patients, the administration of the TNF-α inhibitor infliximab did not report either clinical improvement after its use or significant differences in gut microbiota composition [340]. Despite not exerting a direct anti-inflammatory but an antioxidant effect, N-acetylcysteine (NAC) has also proven potential benefits for patients with BD, especially as a coadjutant for ameliorating depressive symptoms [333, 341]. Likewise, according to previous studies, the use of NAC alleviates gut dysbiosis and glucose disturbances in mice fed with high-fat diet [342], denoting the potential benefits that this drug may have on gut microbiota and BD.

#### Other lifestyle interventions

Active lifestyle approaches with regular physical activity and sleep hygiene as well as psychosocial interventions are prominent coadjutants to pharmacotherapy [343]. Regarding physical activity levels, patients with BD appear to be less active and more sedentary than the general population [344]. Exercise also displays pleiotropic effects in the organism, including favorable effects on gut microbiota, including the augmentation of the number of beneficial bacteria, microbial diversity while improving the functioning of the whole MGB axis [345]. Because of that, compelling evidence is supporting the role of exercise in patients with BD, improving health measures including depressive symptoms, functioning and quality of life [346]. However, there is still a lack of studies that do not permit to establish a cause-effect relationship between mood and physical exercise and further research is needed to determine the recommended intensity, duration and frequency of exercise programs [347]. An interesting approach could be to study changes in gut microbiota composition before and after following an adapted physical activity training program and possible implications in patients with BD, aiding to understand the effects derived from this intervention.

On the other hand, irregular circadian patterns can promote mania and depression episodes, and potential interventions to ameliorate this disruption have been explored [46]. Aberrant light cycles can modify gut microbiota composition, altering especially relative abundance of Lactobacillus and Bacteroidetes [348]. In this way, novel approaches have been proposed, such as light and social rhythm therapies, which aim to regulate everyday activities, to enhance social relationships and, consequently, reduce the effect of circadian patterns disturbances [46]. These approaches showed to modify gut microbiota in a beneficial way [349] and it seems to be helpful in BD [350], but the capacity of ameliorate mood symptoms and preventing episodes is yet uncertain [351]. Similarly, midday bright light has been proposed, which has already shown efficacy in increasing remission rates in BD [352]. In relation to this fact, not only circadian but also seasonal variations appear to have a major effect on BD patients. Indeed, it seems that there is a peak in hypomanic/manic symptoms in the spring and early summer months versus a peak in depressive symptoms in the late fall and early winter; and these observations may correspond to seasonal changes in solar insolation, alterations in melatonin production and seasonal influence on the hypothalamic–pituitary–thyroid (HPT) axis, especially at locations of increasing distance from the equator [353]. Gut microbiota is equally affected by seasonal variations. For instance, increased levels of Actinobacteria and Firmicutes to Bacteroidetes ratio can be observed in summer [354], partially explained by dietary fluctuations across seasons [355]. Thus, it would be interesting to research if there is a specific relationship between seasonal variations in gut microbiota with the onset and/or switch of different episodes in patients with BD, with a special focus on dietary variations in these patients.

Some studies have reported associations between the menstrual cycle and BD, particularly in a subgroup of women with enhanced hormonal sensitivity, who seem to experience menstrual cycle effects on depressive, hypomanic, and manic episodes. These phase-episode effects appear to be heterogeneous and may have implications for treatment [356]. Although some studies have failed to find these associations [357, 358], a recent narrative review on this topic [359] supports that, despite there are little works in the field (only 22), certain women with BD may have their mood shifts affected by menstrual cycle events, with different patterns according to the type of BD. The co-occurrence of BD with menstrual cycle-related disorders such as premenstrual dysphoric disorder (PMDD) appears to have a noteworthy impact on these patients, leading to greater chronobiological disruptions across the follicular and luteal phases of the menstrual cycle [360]. In this sense, prior works have related menstrual cycles variations with gut microbiota, and nowadays it is widely accepted that there are certain microorganisms directly implicated in the metabolism and effects mediated by estrogens and sex hormones, conforming what is designed as the estrobolome [361]. There are no studies evaluating the role of the estrobolome in women with BD yet, and how variations in these microorganisms can be important in this group. However, deepening on this field could be specially beneficial for these patients, aiding to develop specific interventions to ameliorate the effects of menstrual cycle in mental disorders [362].

Notwithstanding current therapies reduce rates of relapse in BD patients, these effects might not be translated into an increased quality of life for the patient, also neglecting the impact of BD on suicidal thoughts, besides actions and functional outcomes [363]. In this sense, it is important to find personalized therapies using multidisciplinary approaches that fit patients’ needs [364, 365]. For this reason, understanding the role of gut microbiota in BD pathology is extremely important, as this may aid to identify novel therapeutic targets for maximizing the clinical management of these patients. The main findings about translational approaches reviewed in this section are summarized in Table 2.

**Table 2:** Translational approaches modulating gut microbiota in patients with BD.

Adjuvant approach	Therapeutic effect	Preliminary evidence in BD	Main findings on gut microbiota	References
Dietary interventions (Mediterranean diet)	Pleiotropic actions	Patients with BD frequently present lower Mediterranean Diet adherence and evidence of malnutrition (insulin resistance, higher levels of fasting triglycerides, glucose index and waist circumference) Diet modulates multiple biological mechanisms involved in BD, possibly influencing the clinical course and therapy success Mediterranean diet appears to decrease systemic inflammation Diet should be considered as an important coadjutant to pharmacotherapy	Increased SCFA and *Faecalibacterium* Increased gene bacterial gene richness Decreased *Ruminococcus* strains	[275, 279, 282–285, 288]
Omega-3 PUFA	Anti-inflammatory; antidepressant; pleiotropic actions	Improve depressive manifestations in BD, but not manic symptoms and seems to attenuate variability in mood, energy, irritability, and pain	Omega-3 PUFAs modify gut microbiota diversity by increasing the abundance of Bifidobacteria and Akkermansia as well as lowering Enterobacteria abundance Increase of Lactobacillus and Butyrivibrio, together with restoration of Firmicutes/Bacteroidetes ratio	[290–292]
Probiotics/psychobiotics and prebiotics	Health benefits; anti-inflammatory; decrease some risk factors for cardiovascular disease, enhance satiety and promote weight loss	Pro and prebiotics seem to have anxiolytic and antidepressant effect. Its usefulness in BD are promising, but still controversial	Probiotics are live microorganisms (generally Lactobacillus and Bifidobacterium strains) which can interact with gut microbiota, whereas prebiotics induce changes in the composition and/or activity in the gut microbiota, resulting in critical metabolites and products	[79, 295–298, 300, 301, 304, 306]
Postbiotics (SCFA)	Anti-inflammatory and immunomodulatory	SCFAs diminish inflammatory response and apoptosis In rats, sodium butyrate showed to have an antimanic effect SCFA seems to be associated with BD symptomatology	SCFA could have an epigenetic activity and they may modulate histone acetylation and NF-κB signaling	[205, 207, 311–313]
Antimicrobials (tetracyclines, i.e., minocycline and doxycycline)	Antibiotic and antidepressant effect	Anti-inflammatory and neuroprotective activity. Tetracycline antibiotics might have a lithium-like effect and an antidepressants activity Minocycline may be useful for patients with high GSH levels Aspirin and minocycline combination shown to be effective in BD depression	Minocycline modulates glutamate and monoaminergic pathways. It also Reduces gut microbiota diversity and reduces the Firmicutes/Bacteroidetes ratio Decreases Allobaculum, *Bifidobacterium*, *Turicibacter* and *Clostridium* genera. In humans, it lowers *Lactobacillus salivariu*s, *Bifidobacterium adolescentis* and *Bifidobacterium breve* strains Increases *Akkermansia*, *Lachnospiraceae* and *Ruminococcaceae incertae sedis* Doxycycline drives to a short-term reduction of lactic acid bacteria like Bifidobacterium or Lactobacillus	[316–319, 323–325]
Fecal microbiota transplantation (FMT)	Gut microbiota restoration	A woman diagnosed with BD went through FMT from her healthy husband at least nine times. Afterward, she had neither manic nor depressive symptoms in the following sixth months, also presenting an important weight loss of nearly 33 kg	Possible beneficial changes of gut microbiota according to the healthy donor	[329, 331]
Immune-based approaches	Anti-inflammatory; antidepressant effects	Celecoxib reduces anxiety and accelerates treatment response in BD TNF-α inhibitors have also shown antidepressant properties NAC can be beneficial for alleviating depressive symptoms in patients with BD	Celecoxib does not seem to alter bacterial abundance, but relative changes in some bacterial populations have been described and it reduces butyrate production The administration of infliximab did not show significant differences in gut microbiota composition NAC alleviates gut dysbiosis and alterations in glucose metabolism in mice	[333, 334, 336–338, 340–342]
Physical activity	Pleiotropic effects	Patients with BD are less active and more sedentary than general population Physical activity can be a promising coadjutant to pharmacotherapy in BD patients	Physical activity widely increases bacterial diversity and beneficial bacteria	[344–347]
Light therapy and social rhythm therapy	Mood stabilizer; improves quality of life	Increases remission rates in BD. Clinical benefits have been reported, but the capacity of ameliorate mood symptoms and preventing episodes is yet uncertain	Beneficial changes in gut microbiota have been reported. Increases in *Akkermansia muciniphila*, *Bifidobacterium sp*., and *Faecalibacterium sp* Decreases in the *Firmicutes:Bacteroides* ratio	[350–352]

## Conclusions

The dialog between MGB axis and immune system represents a crucial biological mechanism in BD, being tightly linked to other pathophysiological events. There seems to be an interplay within aberrant neurotransmission, chronic low-grade inflammation and host metabolic state that would explain characteristic mood fluctuations in this complex and incapacitating disorder. In our search for evidence about BD-gut microbiota, we realized that there seem to be more studies related to depressive symptoms and changes in the dynamism of the gut microbiota (different concentrations of SCFAs or relative bacterial abundances). Hence, it would be equally important to deepen investigations related to other clinical phases like hypomania/mania and mixed features, also differencing between type I and type II BD. Pharmacological treatment of BD, which is of great importance in the clinical management of BD, has also critical effects on gut microbiota, opening the opportunity to use this knowledge as potential biomarkers. Finally, the very probable involvement of gut microbiota in the pathogenesis of this psychiatric disorder opens promising approaches to modulate the gut microbiota and the MGB axis, including dietary interventions, the use of pre-, pro- and postbiotics and other therapeutic approaches like FMT and other lifestyle interventions. Overall, the MGB axis represents an additional but a critical point of study in the complex field of BD and deepening on this exciting line of research could allow the development of promising translational approaches together with better monitoring of BD individuals, potentially improving the clinical management and quality of life of these patients.
